# miR‐200a/b/‐429 downregulation is a candidate biomarker of tumor radioresistance and independent of hypoxia in locally advanced cervical cancer

**DOI:** 10.1002/1878-0261.13184

**Published:** 2022-02-15

**Authors:** Anja Nilsen, Tiril Hillestad, Vilde E. Skingen, Eva‐Katrine Aarnes, Christina S. Fjeldbo, Tord Hompland, Tina Sandø Evensen, Trond Stokke, Gunnar B. Kristensen, Beata Grallert, Heidi Lyng

**Affiliations:** ^1^ Department of Radiation Biology Norwegian Radium Hospital Oslo University Hospital Norway; ^2^ Department of Core Facilities Norwegian Radium Hospital Oslo University Hospital Norway; ^3^ Department of Gynecological Oncology Norwegian Radium Hospital Oslo University Hospital Norway; ^4^ 6305 Institute of Cancer Genetics and Informatics Oslo University Hospital Norway; ^5^ 6305 Department of Physics University of Oslo Norway

**Keywords:** central pelvic recurrence, cervical cancer, extracellular matrix interaction, microRNA, miR‐200, radioresistance

## Abstract

Many patients with locally advanced cervical cancer experience recurrence within the radiation field after chemoradiotherapy. Biomarkers of tumor radioresistance are required to identify patients in need of intensified treatment. Here, the biomarker potential of miR‐200 family members was investigated in this disease. Also, involvement of tumor hypoxia in the radioresistance mechanism was determined, using a previously defined 6‐gene hypoxia classifier. miR‐200 expression was measured in pretreatment tumor biopsies of an explorative cohort (*n* = 90) and validation cohort 1 (*n* = 110) by RNA sequencing. Publicly available miR‐200 data of 79 patients were included for the validation of prognostic significance. A score based on expression of the miR‐200a/b/‐429 (miR‐200a, miR‐200b, and miR‐429) cluster showed prognostic significance in all cohorts. The score was significant in multivariate analysis of central pelvic recurrence. No association with distant recurrence or hypoxia status was found. Potential miRNA target genes were identified from gene expression profiles and showed enrichment of genes in extracellular matrix organization and cell adhesion. miR‐200a/b/‐429 overexpression had a pronounced radiosensitizing effect in tumor xenografts, whereas the effect was minor *in vitro*. In conclusion, miR‐200a/b/‐429 downregulation is a candidate biomarker of central pelvic recurrence and seems to predict cell adhesion‐mediated tumor radioresistance independent of clinical markers and hypoxia.

## Graphical abstract

Diagnostic biomarkers for identifying tumor radioresistance in patients with cervical cancer are highly needed. Here, we report a new candidate biomarker based on the miR‐200a/b/‐429 cluster expression in tumor biopsies. Low expression of miR‐200a/b/‐429 in patients was found to predict the risk of central pelvic recurrence after chemoradiotherapy. Additionally, miR‐200a/b/‐429 overexpression radiosensitized tumor xenografts. Moreover, the expression of the miR‐200a/b/‐429 was independent of known risk factors and was predictive of cell adhesion‐mediated radioresistance in patients.

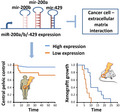

**Abbreviations**
ADC: apparent diffusion coefficients
CI: confidence interval
Ct: threshold cycle
DSB: DNA double strand break
DW‐MRI: diffusion weighted magnetic resonance imaging
ECM: extracellular matrix
EMT: epithelial to mesenchymal transition
FDR: false discovery rate
FIGO: Federation International de Gynecologie et d'Obstetrique
GEO: Gene Expression Omnibus
GO: gene ontology
HR: hazard ratio
KEGG: Kyoto Encyclopedia of Genes and Genomes
miRNA: microRNA
MR: magnetic resonance
N.S: nonsignificant
RECIST: response evaluation criteria in solid tumors
REMARK: reporting recommendations for tumor marker prognostic studies
ROI: region of interest
SEM: standard error of mean

## 1 Introduction

Radiotherapy, most often combined with cisplatin, is the treatment of choice for patients with locally advanced cervical cancer [1, 2, 3]. Improved treatment strategies are highly needed, since cancer recurrence after therapy is seen in about 30% of the patients, and in almost 65% at the more advanced stages. Many patients develop a highly radioresistant tumor, and more than 25% of all recurrences are located within the radiation field [1, 2, 3]. These patients have limited further treatment options [4, 5]. A better understanding of the molecular mechanisms behind tumor radioresistance in cervical cancer can lead to new therapeutic approaches [6]. Moreover, such knowledge would help development of biomarkers for identifying patients at risk of recurrence in the radiation field, as current clinical markers like tumor stage, tumor size and lymph node status are insufficient for this purpose [7].

microRNAs (miRNAs) are important regulators of biological processes affecting the response of tumor cells to radiation, including DNA damage repair, hypoxia tolerance, cell survival, and proliferation [8]. The small, noncoding RNAs downregulate expression of their target genes by degrading mRNAs and inhibiting translation. They have a large potential as biomarkers due to high stability in tissue specimens and biofluids [9], but no candidates for radioresistance have yet been evaluated in a decent number of cervical cancer patients. A comprehensive molecular characterization of 228 tumors of patients mainly treated with surgery identified downregulation of miR‐200a and miR‐200b and upregulation of some of their target genes as frequent events in cervical cancer [10]. These miRNAs therefore seem to be functionally active and promising candidates for further exploration in patients receiving chemoradiotherapy.

The miR‐200 family consists of nine mature miRNAs in two clusters located on chromosome 1 (miR‐200a‐5p, miR‐200a‐3p, miR‐200b‐5p, miR‐200b‐3p, and miR‐429) and chromosome 12 (miR‐200c‐5p, miR‐200c‐3p, and miR‐141‐5p miR‐141‐3p) [11]. miR‐200a has been included in two prognostic miRNA‐signatures in cervical cancer [12, 13], but the role of the miR‐200 family in tumor radioresistance has not been addressed in this disease. The members regulate large networks of genes that combined suppress various biological processes including epithelial to mesenchymal transition (EMT) and are often found to be downregulated in cancer [11]. EMT involves interaction between the extracellular matrix (ECM) components collagens and glycoproteins, and cell surface molecules like integrins and growth factor receptors. Such interactions regulate several downstream pathways that have been implicated in the radiation response of tumor cells [14, 15]. Moreover, members of the miR‐200 family have been found to be downregulated by hypoxia [16, 17, 18], which is a tumor feature associated with failure of chemoradiotherapy in cervical cancer [19, 20, 21]. It is therefore likely that the miR‐200 family plays a role in the radioresistance of this disease.

The recurrence pattern of cervical cancer patients receiving chemoradiotherapy includes distant metastases and locoregional recurrence within the pelvic radiation field [4]. A higher radiation dose is given centrally to the tumor region than to the lateral part of the pelvis. A distinction between central and lateral pelvic recurrence is therefore needed to identify radioresistant tumors. In this work, we assessed a unique set of site‐specific recurrence data of 200 patients, as well as expression data of the miR‐200 family and their predicted target genes for all tumors. Our aim was to investigate whether members of the miR‐200 family play a role in the radioresistance of cervical tumors and may be potential biomarkers of central pelvic recurrence after chemoradiotherapy. We further aimed to clarify whether hypoxia, as assessed by a 6‐gene hypoxia classifier from previous work [19], was involved in this resistance mechanism. Proof‐of‐principle radiation experiments in a cervical cancer model system with overexpressed miRNAs were performed to validate our conclusion from the clinical analyses. Our results provide novel insight into a role of the miR‐200 family in cervical cancer radioresistance that is independent of tumor hypoxia and may be exploited for a more precise identification of patients with risk of central pelvic recurrence.

## 2 Materials and methods

### 2.1 Patient cohorts

The study included 200 patients with locally advanced squamous cell carcinoma of the uterine cervix enrolled in our prospective chemoradiotherapy protocol at the Norwegian Radium Hospital from 2001 to 2012 (Table S1). Totally 1–4 tumor biopsies were taken at diagnosis, snap‐frozen, stored at −80 °C, and used for miRNA and gene expression analysis. The patients were assigned to two different cohorts based on the Illumina bead array version that was used for gene expression profiling, that is, HT‐12 v4 (explorative cohort, 90 patients) or WG‐6 v3 (validation cohort 1, 110 patients) [19]. Tumor volume, pathologic lymph nodes, and Federation International de Gynecologie et d'Obstetrique (FIGO) stage were determined by magnetic resonance (MR) imaging or computed tomography at diagnosis, according to the REsponse evaluation Criteria In Solid Tumors (RECIST) v1.1guidelines [22].

All patients received external radiotherapy, followed by intracavitary brachytherapy. Concurrent cisplatin (40 mg·m^−2^ weekly) was given according to tolerance. Follow‐up was performed as described [19]. Site‐specific recurrence data were collected and classified as central (pelvic recurrence within the center of the radiation field), lateral (pelvic recurrence within the radiation field outside the central region), and/or distant (metastases outside the radiation field). The research was conducted in accordance with the Declaration of Helsinki. The study was approved by The Regional Committee for Medical and Health Research Ethics in South East of Norway (REC 2016/2179), and written informed consent was obtained from all patients. The study adheres to the REporting recommendations for tumor MARKer prognostic studies (REMARK) guidelines [23].

### 2.2 miRNA profiling and calculation of miR‐200a/b/‐429 score

miRNA expression profiles of the 200 patients in explorative cohort and validation cohort 1 were generated from biopsies with a tumor cell fraction above 50%. The tumor cell fraction was estimated by a person experienced in cervical cancer pathology. This estimation was based on visual inspection of a hematoxylin‐ and eosin‐stained histological section from the biopsy. Total RNA from samples in the explorative cohort was isolated from frozen sections, using miRNeasy kit (Qiagen, Hilden, Germany) according to the manufacturer's protocol. Total RNA from samples of validation cohort 1 was isolated from frozen sections, using TRIzol^®^ Reagent (Thermo Fisher Scientific, Waltman, MA, USA) followed by double precipitation with isopropanol, according to the manufacturer's recommendation. RNA was further purified by precipitation with 5 m lithium chloride and washed with 70% ethanol. Following either RNA isolation protocols, the RNA pellet was dissolved in RNase‐free water and stored at −80 °C until labeling. RNA concentration was measured by a NanoDrop 1000 spectrophotometer (NanoDrop Technologies, Wilmington, DE, USA). The RNA integrity was assessed by Bioanalyzer 2100 (Agilent Technologies, Santa Clara, CA, USA), and samples with RNA integrity number above 6.5 were used. The stability of miRNAs in stored tissue samples was assessed in a previous study and showed no degradation during storage time [24]. RNA of different samples from the same tumor was pooled.

Small RNA preparation for sequencing was performed according to Illumina TruSeq Small RNA library protocols (Illumina Inc., San Diego, CA, USA). In brief, indexed 3′‐ and 5′‐end RNA adapters were ligated to isolated total RNA (1 μg) per sample followed by reverse transcription and library amplification. The cDNA was purified by cutting out bands corresponding to 140–160 bp (length of miRNA + adapter and index sequences) on a PAGE gel, pooling, and precipitation with ethanol. Single‐end sequencing of the cDNA molecules was performed on the Illumina HiSeq 2500 platform. Real‐time analysis, base calling, and filtering of low‐quality reads were performed by Illumina's software packages (Illumina Inc.). fastq data were quality checked, and reads were aligned, quantified, and annotated using the miRDeep2 algorithm with miRBase v21 for annotation of mature miRNAs [25]. Reads per million annotated mature miRNAs were used for normalization of read counts. Log_2_‐transformed data were used in the analyses. Data have been deposited in the Gene Expression Omnibus (GEO), accession number GSE178629.

A miR‐200a/b/‐429 score was calculated for each patient by averaging the median centered, log_2_‐transformed expression levels of miR‐200a‐5p, miR‐200a‐3p, 200b‐5p, miR‐200b‐3p, and miR‐429.

### 2.3 External miRNA data set

Publicly available miRNA data of a third cohort (*n* = 79 tumor samples, validation cohort 2) were included for the validation of prognostic significance. The cohort included patients treated with radiation and concurrent cisplatin at Princess Margaret Cancer Centre in Toronto, Canada, between 2000 and 2007. Progression‐free survival and miRNA data were available for the ‘training cohort’ (‘TLDA data, Training cohort’) in the publication by How et al. [26].

miRNA data were derived from snap‐frozen biopsies taken at diagnosis, as described [26]. Total RNA was isolated from frozen biopsies with a tumor cell fraction above 70%, using Norgen Total Purification kit (Norgen Biotek, Thorold, ON, Canada). miRNA expression profiles were measured with RT‐qPCR array, using the TaqMan Low Density Array (TLDA) Human MicroRNA A Array v2.0 and the 7900HT Real‐Time PCR System (Applied Biosystems, Carlsbad, CA, USA). miRNA expression levels were calculated using the mean threshold cycle (Ct) of the three control RNAs RNU44, RNU48 and U6 for normalization. None of the miRNAs had any Ct‐values above 37. A miR‐200a/b/‐429 score was calculated as described in Section 2.2, but based on only miR‐200a‐3p, miR‐200b‐3p, and miR‐429, since data of miR‐200a‐5p and miR‐200‐5p were not available.

### 2.4 Gene expression profiling and hypoxia status

The gene expression data and hypoxia status of all patients in the explorative cohort and validation cohort 1 are parts of a data set used in previous work [19] and are available in GEO (GSE72723). In brief, gene expression profiling of fresh‐frozen tumor biopsies was performed using Illumina bead array versions HT‐12 v4 (explorative cohort) and WG‐6 v3 (validation cohort 1) with 47 231 and 48 803 transcripts, respectively (Illumina Inc.). The same RNA isolation protocols that were used for miRNA profiling were applied for each sample. Signal extraction and quantile normalization were performed using the software provided by the manufacturer (Illumina Inc.), and log_2_‐transformed gene expression data were used in the analyses [19]. A 6‐gene hypoxia signature was derived from the gene expression data of *ERO1A, DDIT3, KCTD11, P4HA2, STC2,* and *UPK1A*, as described in previous work [19]. The signature has a predefined cutoff of zero, which was used for dichotomous classification of tumors according to their hypoxia status as more or less hypoxic.

### 2.5 Target gene prediction

Candidate target genes for miR‐200a‐5p/‐3p, 200b‐5p/‐3p, and miR‐429 were collected from the miRGate database, including both validated and computationally predicted targets from several databases [27]. The expression values of the candidates were correlated with the corresponding miRNA expressions of the explorative cohort and validation cohort 1 separately, using Spearman correlation analysis (two‐sided) with the Benjamini–Hochberg procedure [28] to control the false discovery rate (FDR). Genes with a significant negative correlation (nominal *P* < 0.05 in both cohorts and FDR *q*‐value < 0.1 in at least one cohort) were considered as potential target genes. These requirements were used to compromise between the risk of losing true discoveries and the risk of keeping noisy, irrelevant data. The result was visualized in a regulatory network with miR‐200a‐5p/‐3p, 200b‐5p/‐3p, and miR‐429 as nodes and their potential target genes as interaction partners. Interactions with an FDR *q*‐value < 0.1 were included in the network. The network was created by the cytoscape software v3.6.1 [29].

### 2.6 Generation of SiHa cell line with stable miR‐200a/b/‐429 overexpression

miRNA overexpression rather than repression was chosen due to low endogenous expression of the miR‐200 family in cervical cancer cell lines. In accordance with the histology of the clinical tumor samples (Table S1), the cervical squamous cell carcinoma cell line SiHa (ATCC^®^ HTB‐35™ from ATCC, LGC standards, Wesel, Germany) was used. Cell line authentication was performed with Powerplex 16 (Promega, Madison, WI, USA) to identify DNA STR profiles. Cells were cultured at 37 °C in DMEM medium containing Glutamax (Gibco, Life Technologies, Carlsbad, CA, USA) supplemented with FBS (10%) and PenStrep (1%) in 5% CO_2_ and 95% room air. All cell line experiments were performed between passage two and 14. Mycoplasma testing was conducted at a regular basis with MycoAlert™ Mycoplasma Detection Kit (Lonza, Cologne, Germany).

A stably expressed miR‐200a/b/‐429 SiHa cell line and a control SiHa cell line (empty vector), both with co‐expressed GFP, were generated by lentiviral transduction. pLenti 4.1 Ex miR200b‐200a‐429 GFP (Addgene plasmid #35533; Addgene, Cambridge, MA, USA) was a gift from G. Goodall [30]. Empty vector control plasmid, pLenti 4.1 Ex GFP was derived from pLenti 4.1 Ex miR200b‐200a‐429 GFP by removal of the miRNA precursor sequence with the restriction enzymes Nhe1 (R3131S; New England Biolabs, Ipswich, MA, USA) and AvrII (R0174S; New England Biolabs). Lentivirus was generated by co‐transfection of packaging plasmids and the pLenti 4.1 Ex miR200b‐200a‐429 GFP or pLenti 4.1 Ex GFP (control) construct into HEK293T cells. The 293T cells produced lentiviral particles for 3 days before isolation from the culture medium. SiHa cells were infected with the generated lentivirus. Stably transduced cells (SiHa mir‐200b‐200a‐429‐GFP or SiHa control‐GFP) were selected by culturing in puromycin (1 μg·mL^−1^) for 20 days. Subsequent cell culturing conditions were according to common practice, using DMEM medium containing Glutamax (Gibco) supplemented with FBS (10%) and PenStrep (1%) in 5% CO_2_ and 95% air.

Stably transduced cells were further selected based on GFP intensity by cell sorting, using a SH800 Cell Sorter (Sony, Tokyo, Japan), and based on expression level of miR‐200a‐5p/‐3p, miR‐200b‐5p/‐3p and miR‐429 measured by RT‐qPCR. For RT‐qPCR analysis, total RNA was isolated with the miRNeasy kit (Qiagen), dissolved in nuclease‐free water, and stored at −80 °C prior to use. RNA concentration was measured on a NanoDrop 1000 spectrophotometer (NanoDrop Technologies). cDNA was synthesized from 10 ng of total RNA using the miRCURY LNA™ microRNA PCR, Polyadenylation, and cDNA synthesis kit II (Qiagen). Predeveloped microRNA LNA™ PCR primer sets (Qiagen) were used for amplification of the selected miRNAs: hsa‐miR‐200a‐3p; (prod. no. 204707), hsa‐miR‐200a‐5p (prod. no. 206063), hsa‐miR‐200b‐3p (prod. no. 2060071), hsa‐miR‐200b‐5p (prod. no. 204144), and hsa‐miR‐429; (prod.no. 205901). Hsa‐miR‐151a‐5p (prod. nr 204007) and hsa‐miR‐152‐3p (prod. nr 204294) were used as reference miRNAs for normalization of miRNA expression in the RT‐qPCR analysis, based on our previous work [24]. The miRCURY Universal RT miRNA PCR system (Qiagen) and Bio‐Rad Real‐Time PCR thermocycler (Bio‐Rad, Hercules, CA, USA) were used for amplification and detection of miRNAs according to manufacturer's protocols. Amplification was analyzed using the sds Software v2.3 (Applied Biosystems). Reverse transcriptase negative controls and nontemplate controls were included and showed no amplification. Relative miRNA expression level was calculated using the comparative ΔCt (cycle threshold) method by normalizing the Ct value of each miRNA to the mean Ct value of the reference miRNAs: ΔCt_miRNA_ = Ct_miRNA_ − (Ct_miR‐151a‐5p_ + Ct_miR‐152‐3p_/2). A Z2 Coulter particles counter and size analyzer (Beckman Coulter, Brea, CA, USA) with a 100‐μm capillary was used to measure cell proliferation and cell size, respectively.

### 2.7 Western blotting

Protein extracts from cell lines were prepared by lysing the samples in RIPA‐buffer (Thermo Fisher Scientific) containing Halt™ Phosphatase Inhibitor Cocktail and Halt™ Protease Inhibitor Cocktail EDTA‐Free (Thermo Fisher Scientific). Lysates were incubated for 30 min on ice followed by centrifugation for 15 min at 16 000 ***g***. The supernatants were added Pierce Lane Marker Reducing Sample Buffer (Thermo Fisher Scientific) and boiled at 95 °C for 5 min. The protein extracts (20 μg) were electrophoresed in a Mini‐PROTEAN^®^ TGX Stain‐Free™ Protein Gel (Bio‐Rad) and transferred to a Trans‐Blot Turbo RTA Mini 0.2 μm PVDF membrane (Bio‐Rad). Mouse anti‐E‐cadherin monoclonal antibody (#610181; BD Biosciences, San Jose, CA, USA) was used as primary antibody and diluted 1 : 5000 in PBS with 5% dry milk. HRP‐conjugated polyclonal donkey anti‐mouse‐IgG was used as secondary antibody (715‐035‐150; Jackson Immunoresearch, West Grove, PA, USA). The blot was imaged in a Chemidoc MP (Bio‐Rad) using LumiGlo (KPL, Gaitersburg, MD, USA) substrate for detection. Total protein was used as loading control and detected with Stain‐Free technology imaging (Bio‐Rad) of the membrane after transfer.

### 2.8 Clonogenic survival assay of irradiated cells

miR‐200a/b/‐429‐overexpressing cells or control cells were grown to 60–70% confluence and exposed to 4 or 8 Gy of ionizing radiation in single fractions at a dose rate of 1.0 Gy·min^−1^ with a 160 kV X‐ray generator (Faxitron Cabinet X‐ray; Faxitron, Tucson, AZ, USA). Cells were immediately trypsinized (0.05% trypsin; Sigma‐Aldrich, St. Louis, MO, USA), diluted, and seeded into 60‐mm culture dishes for clonogenic survival assays. After 14 days in culture, cells were fixed in ethanol (70%) and stained with methylene blue (0.5%). Colonies with > 50 cells were counted, and survival fraction was calculated. Plating efficiencies were calculated from nonirradiated cells.

### 2.9 Cell cycle distribution and DNA double strand break (DSB) detection by flow cytometry

miR‐200a/b/‐429‐overexpressing cells or control cells were cultured until they reached 60–70% confluence and irradiated with 8 Gy at a dose rate of 1.0 Gy·min^−1^. Samples were collected at the timepoints 0, 0.5, 1, 2, 6, 24, and 48 h after radiation, incubated with LIVE/DEAD™ Fixable Near‐IR Dead Cell Stain (L34975; Life Technologies AS, Carlsbad, CA, USA) on ice for 30 min, fixed in ice cold ethanol (70%), and stored at −20 °C before analyses.

About 1 × 10^6^ cells of each sample were added 2.5 × 10^5^ ethanol fixed lymphocyte leukemia REH cells. The REH cells were applied in a 1 : 1 mixture of irradiated (4 Gy) and nonirradiated cells and were used for normalization to reduce effect of intersample staining variations. The samples were rehydrated in PBS and incubated for 30 min in room temperature with the DSB marker mouse anti‐γH2Ax (#05‐636; Millipore, Burlington, MA, USA) and rabbit anti‐phospho‐histone H3 (Ser10) mitosis marker (#06‐570; Millipore) diluted 1 : 500 in PBS with 5% nonfat milk. The samples were further washed in PBS, incubated for 30 min in room temperature with the secondary antibodies goat anti‐mouse IgG Alexa Fluor 594 (ab150116; Abcam, Cambridge, MA, USA) and donkey anti‐rabbit IgG AlexaFluor 647 (A31573; Thermo Fisher Scientific), washed in PBS, and resuspended in PBS containing 1.5 μg·mL^−1^ Hoechst 33258 (Sigma‐Aldrich) for DNA staining. They were thereafter run on a LSR II yellow laser flow cytometer (BD Biosciences) and analyzed with flowjo software (v10; TreeStar Inc., San Jose, CA, USA).

Gating of cell populations is illustrated in Fig. S1. The whole population of SiHa cells stained with LIVE/DEAD Near‐IR was distinguishable from unstained REH cells with the red laser (640 nm). The median γH2Ax intensity of nonmitotic, live miR‐200a/b/‐429‐overexpressing cells or control cells was normalized to the median γH2Ax intensity of the REH population in the same sample.

### 2.10 Generation of SiHa xenograft tumors with stable miR‐200a/b/‐429 overexpression

Tumor xenografts with stable miR‐200a/b/‐429 overexpression were established in female athymic nude mice (Hsd:Athymic Nude‐Foxn1nu; originally supplied by Envigo, Indianapolis, IN, USA). The mice were bred in‐house at Department of Comparative Medicine at our institution and kept in specific pathogen‐free environment with food and water supplied *ad libitum*, 12‐h light‐dark cycle, temperature of 22 ± 1 °C and 65 ± 5% humidity. Mice were monitored at daily base. All procedures were conducted according to the European Laboratory Animal Science Association (FELASA) regulations, and studies were approved by the Norwegian Food Safety Authority (FOTS #8344).

At an average age of 6–7 weeks, the mice were injected with miR‐200a/b/‐429‐overexpressing cells or control cells (2 × 10^6^) intramuscularly in both hind legs for characterization studies of unirradiated tumors. Tumor growth was monitored by anatomical T_2_‐weighted (T_2_W) MR imaging (MRI) at regular timepoints. Tumor doubling time (Td) was calculated for each tumor in the exponential growth phase and fitted to an exponential growth regression model. The curves were used to calculate mean Td for each group of tumors. At the day of tumor resection, diffusion weighted (DW)‐MRI was used to assess tumor apparent diffusion coefficients (ADC) as a measure of cell density. The median tumor volume was 252 mm^3^. The hypoxia marker pimonidazole (60 mg·kg^−1^; Hypoxyprobe Inc., Burlington, MA, USA) was administered intraperitoneally 90–120 min before the mice were euthanized by cervical dislocation and tumors were resected, paraformaldehyde‐fixed (4%), and paraffin‐embedded for digital histopathology.

### 2.11 Radiation experiments on xenograft tumors

For radiation experiments on xenografts, mice at an age of 6–7 weeks were injected with miR‐200a/b/‐429‐overexpressing cells or control cells intramuscularly in the right hind leg. At a tumor volume of 200–250 mm^3^, the mice were anesthetized by subcutaneous injection with a mixture of 10–15 mg·kg^−1^ xylasin (Rompun^®^; Bayer AG, Leverkusen, Germany), 5–10 mg·kg^−1^ butorphanol (Torbugesic^®^; Zoetis, Berlin, Germany), and 15–20 mg·kg^−1^ zolazepam and tiletamine (Zoletil^®^; Virbac, Carros, France) before irradiation with a single dose of 8 Gy (1.6 Gy·min^−1^) from an X‐ray source (Faxitron MultiRad 225; Faxitron). Postirradiation tumor growth and ADC values were assessed once every 3 or 4 days by T_2_W‐ and DW‐MRI, respectively. Mice were sacrificed by cervical dislocation when the tumor volume had reached at least two times the volume at baseline (day 0), or at latest 82 days after inoculation.

### 2.12 Magnetic resonance imaging

Magnetic resonance imaging of xenograft tumors was conducted with a Bruker Biospec 7.05 T bore magnet (Bruker Biospin AG, Fallanden, Switzerland) with a mouse quadrature volume coil. Animals were anesthetized with a continuous supply of sevoflurane (3–4% in O_2_; Baxter, Guyancourt, France). The body core temperature was monitored and kept at 37 °C during scanning.

To assess tumor volume, anatomical T_2_W images were acquired with a fast spin echo pulse sequence, using an echo time of 31.05 ms, field of view of 3 × 3 cm, matrix of 256 × 256, slice thickness of 0.8 mm, and repetition time of 3000 ms. DW‐MR images for ADC measurement in the characterization studies were acquired with DW single‐shot fast spin echo sequence (field of view of 3 × 3 cm; matrix of 64 × 64; slice thickness of 0.8 mm; repetition time of 1100 ms; echo time of 26 ms; diffusion weightings or *b*‐values of 200, 400, 700 and 1000 s·mm^−2^). DW‐MRI for assessing effect of radiation was acquired with DW echo planar imaging (field of view of 3 × 3 cm; matrix of 128 × 128; slice thickness of 0.8 mm; repetition time of 1500 ms; echo time of 17.89 ms; diffusion weightings or *b*‐values of 200, 300, 500, 700 and 800 s·mm^−2^). ADC values were extracted voxel‐wise from the indicated ranges of *b*‐values, and calculated using in‐house developed scripts in matlab (MathWorks, Natick, MA, USA). This was done by fitting the log‐transformed signal intensities (*S*) to the linear equation ln(*S*(*b*)/*S*0) = −*b*ADC + *c*, using a linear least square fit algorithm. Tumor regions of interest (ROIs) were defined in the T_2_W images and used to construct ADC maps. Areas were defined as necrotic if ADC > 0.0011 mm^2^·s^−1^. The median ADC value of each tumor was used for subsequent analyses.

### 2.13 Digital histopathology

Serial sections (4 μm) were made through the central region of paraffin‐embedded xenograft tumors and prepared for histological staining with standardized protocols using the Dako EnVision™ Flex+ System (K8012; Dako, Carpinteria, CA, USA). Staining with rabbit anti‐GFP (1 : 5000, ab290; Abcam) for detection of transduced tumor cells, rabbit anti‐Ki‐67 (1 : 1000, ab15580; Abcam) for assessment of proliferating cells and necrosis, and rabbit anti‐pimonidazole (1 : 3500, PAb27HAP; Hypoxyprobe Inc.) to quantify hypoxic fraction was performed and visualized using 3,3′‐diamenobendizine (DAB) as chromogen. Hematoxylin was used for counterstaining. Stained tumor sections were digitized (0.46 μm·pixel^−1^, 20× mode) using a whole slide scanner (Nanozoomer‐NZ Digital Slide scanner; Hamamatzu Photonics, Hamamatsu, Japan). Comparable ROI‐boundaries were drawn for adjacent sections stained for different markers and used in the subsequent analyses (Fig. S2).

In‐house developed software programs in matlab (MathWorks) were used for image analyses. Color deconvolution was performed to separate the brown DAB color information in the image, as described [3]. Necrotic regions were defined in the Ki‐67 stained sections by manual outlining of decellularized areas in RGB images (Fig. S3A). Necrotic fraction was calculated as total necrotic area relative to ROI. The necrotic areas were excluded from other analyses. Hypoxia was defined in pimonidazole stained sections. Segmentation and thresholding of pimonidazole‐positive regions were performed as described [31]. Briefly, the hypoxic regions were defined as the regions with an intensity value larger than a manually decided intensity threshold in the DAB‐image (Fig. S3B). Hypoxic fraction was calculated as total hypoxic area relative to ROI after exclusion of necrosis. To quantify cell density, segmentation of GFP‐positive cells was first performed by intensity thresholding of the color deconvoluted DAB image to define tumor parenchyma (Fig. S4). The area of GFP‐positive cells was quantified as total GFP‐positive area relative to ROI after exclusion of necrosis. Cell nuclei were thereafter identified in binary Ki‐67 maps by applying segmentation thresholding on the color deconvoluted DAB images (Fig. S4). All tumor cells were Ki‐67 positive. Binary structures with size > 200 pixels were assumed to be clusters of cells and further divided by applying the Watershed function on the Euclidean distance transformed binary image (matlab). Cells segmented in necrotic areas were removed from the binary map. A binary Ki67‐positive cell mask was further created by including only the segmented cells within the tumor parenchyma (Fig. S4), as defined in the adjacent GFP‐stained section. Cell density was calculated as number of Ki‐67‐positive cell nuclei relative to total GFP‐positive area.

### 2.14 Statistical analysis

All statistical analyses were performed using r [32] v3.5.1, brb‐arraytools v4.5.1 [33], ibm spss v25 (IBM Corp., Chicago, IL, USA), sigmaplot statistical software package v14 (Systat Software Inc., San Jose, CA, USA), x‐tile software v3.6.1 [34], g*power software v3.1.9.7 [35], and david v6.8 web tool [36]. A significance level of *P* < 0.05 was considered statistically significant, if not otherwise stated. Cox uni‐ and multivariate proportional hazards (PH) survival analyses were performed with endpoints central pelvic control, lateral pelvic control, distal control, and progression‐free survival (survival without central, lateral, and/or distal recurrence). Assumptions of PHs in the Cox regression models were evaluated graphically using log‐minus‐log plots. The multivariate analyses were performed by forward and backward stepwise Cox PH regression to test the prognostic and predictive value of the miR‐200a/b/‐429 score, using conventional clinical markers and hypoxia status as covariates. Only the covariates with *P* < 0.1 in univariate analysis were included. Kaplan–Meier survival curves were compared using log‐rank test. Stratification of patients in low or high score groups for these curves was based on the optimal cut‐off for the miR‐200a/b/‐429 score when associated with the different endpoints, as determined by using the x‐tile software.

The functional annotation tool david v6.8 was used to identify enriched gene ontology (GO)‐terms, that is, biological processes, cellular components and molecular functions, and Kyoto Encyclopedia of Genes and Genomes (KEGG) pathways in the list of potential target genes. The global human transcriptome or the total miR‐200a/b/‐429 target gene list collected from the miRGate analysis was selected as background in two separate GO‐ and KEGG analyses. The default GO category ‘GO Direct’ was used for each GO term. The Benjamini–Hochberg procedure [28] was used to control FDR, and FDR *q*‐value < 0.01 was considered statistically significant.

Pearson product moment or Spearman rho two‐sided correlation test was used to search for correlation between parameters. Kruskal–Wallis, chi‐square, Fisher exact, Mann–Whitney *U*‐ or Student's *T*‐test was applied for comparison of groups. The choice between parametric and nonparametric test was based on conditions of normality and type of data (continuous or categorical). Variability of miRNAs over the range of measured values (heteroscedasticity) was checked by visual inspection of plots showing residuals *versus* predicted values and statistically with the White test.

Characterization and radiation response studies on xenografts were performed with 23–30 tumors and 8–10 tumors in each group, respectively. The sample sizes were above the estimated number of tumors needed to detect a significant difference between two groups in a two‐sided Student's *t*‐test with *P* < 0.05, power > 0.8, and 95% confidence interval (CI). These calculations were based on doubling time data of SiHa xenograft tumors from previous work [31]. Endpoint in xenograft tumor growth delay analyses and Kaplan–Meier survival curves was fraction of tumors with a volume below 1.5 times the baseline volume (day 0). An event was defined as the time point when the tumor reached this size (*T*_1.5×_). Matched tumor growth data of nonirradiated xenografts from the characterization experiments were used for comparison. Kaplan–Meier survival curves were compared by Holm–Sidak method for multiple comparisons.

## 3 Results

### 3.1 Expression of miR‐200a/b/‐429 has prognostic impact in three independent cohorts

Expression of the nine miR‐200 members was analyzed in relation to progression‐free survival in the explorative cohort to identify candidates for further analysis. All miRNAs were detected and their expression level varied considerably across the patients (Fig. S5A). Univariate Cox regression analysis revealed a significant association between miRNA expression and progression‐free survival for each of the miR‐200 members on chromosome 1 (miR‐200a‐3p, *P* = 1.3 × 10^−2^; miR‐200a‐5p, *P* = 4.3 × 10^−2^; miR‐200b‐3p, *P* = 4.1 × 10^−2^; miR‐200b‐5p, *P* = 2.4 × 10^−2^ and miR‐429, *P* = 6.7× 10^−3^), where patients with a low expression had poor survival probability compared to the others (Table 1). No prognostic significance was seen for any miR‐200 members on chromosome 12 (miR‐200c‐3p/‐5p and miR‐141‐3p/‐5p).

**Table 1:** Cox regression analyses of progression‐free survival in the explorative cohort (90 patients).

Chromosome 1				Chromosome 12			
miRNA	*P*	HR	95% CI	miRNA	*P*	HR	95% CI
miR‐429	6.7 × 10^−3^	0.40	0.20–0.78	miR‐141‐3p	1.4 × 10^−1^	0.67	0.40–1.14
miR‐200a‐3p	1.3 × 10^−2^	0.44	0.23–0.84	miR‐141‐5p	4.9 × 10^−1^	0.79	0.40–1.56
miR‐200a‐5p	4.3 × 10^−2^	0.52	0.27–0.98	miR‐200c‐3p	3.7 × 10^−1^	0.71	0.33–1.50
miR‐200b‐3p	4.1 × 10^−2^	0.49	0.25–0.97	miR‐200c‐5p	1.5 × 10^−1^	0.65	0.36–1.17
miR‐200b‐5p	2.4 × 10^−2^	0.43	0.21–0.90				

**Fig. 1:**
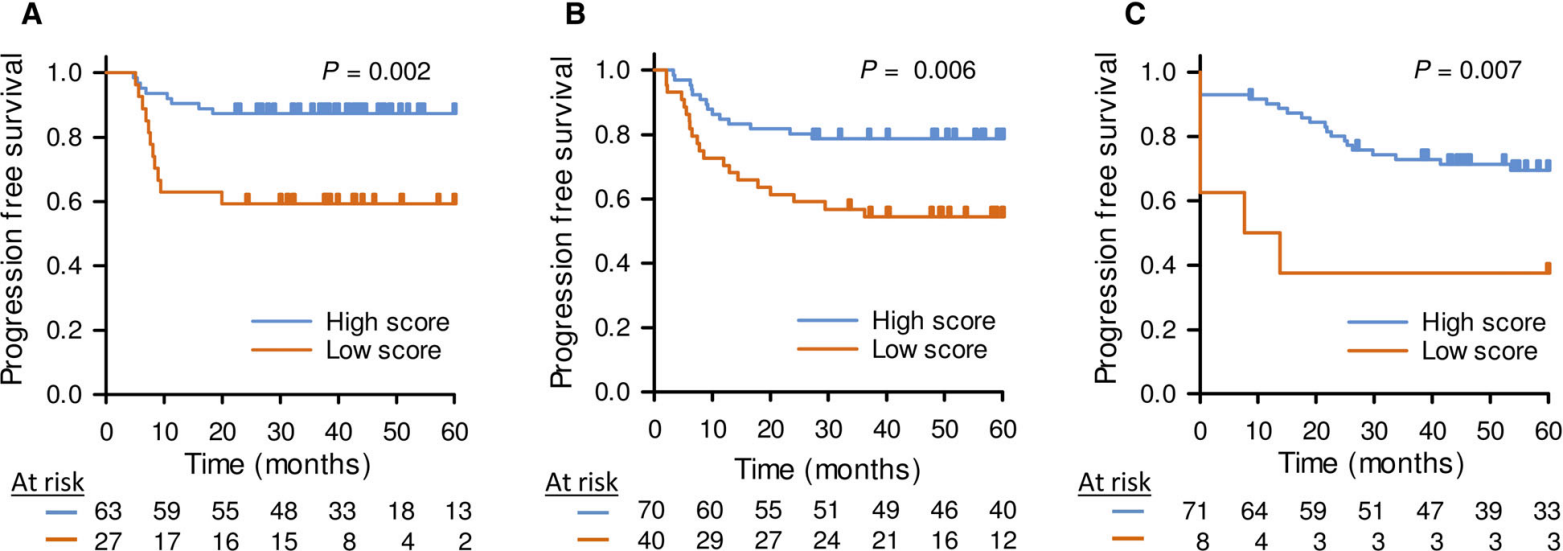
Prognostic significance of the miR‐200a/b/‐429 score in independent cohorts. Kaplan–Meier curves showing progression‐free survival of patients stratified with high‐ and low miR‐200a/b/‐429 score in the (A) explorative cohort (*n* = 90), (B) validation cohort 1 (*n* = 110) and (C) validation cohort 2 (*n* = 79). (A–C) 60 months follow up data were used. *P*‐values from log‐rank test and number of patients at risk are indicated. Patients were divided into a high and low score group based on the strongest association to progression‐free survival.

The miR‐200 members on chromosome 1 were highly co‐expressed, with Pearson correlation coefficients (*r*) in the range of 0.74–0.93 (*P* < 1.0 × 10^−3^ for all; Fig. S5B). Plots of residuals against predicted values showed no patterns of nonconstant variance over the range of measured values for any of the miRNAs, and no significant heteroscedasticity was found (Fig. S6). Since all five mature miRNAs showed prognostic significance and were highly correlated, their expression level was used to calculate a score, termed miR‐200a/b‐429 score for each patient, as outlined in Section 2.2. The score showed prognostic significance when used as a continuous variable [*P* = 1.3 × 10^−2^; hazard ratio (HR), 0.40; 95% CI, 0.19–0.82], consistent with results for the individual miRNAs (Table 1). A cut‐off value that allocated about 30% of the patients to a group with low score, in line with the expected recurrence rate when using progression‐free survival as endpoint, showed the strongest association to outcome in a log‐rank test (Fig. 1A).

The prognostic impact of the miR‐200a/b/‐429 score was further evaluated in validation cohorts 1 and 2. For validation cohort 1, with miRNA sequencing data of patients treated at our institution, a strong association to outcome was found when comparing about 30% of the patients with the lowest miR‐200a/b‐429 score with the others (Fig. 1B). Significant association to outcome was also found in validation cohort 2, with RT‐qPCR miRNA data of patients treated at Princess Margaret Cancer Centre in Toronto, when allocating about 10% of the patients to the high‐risk group (Fig. 1C). These findings validated the prognostic impact of the miR‐200a/b/‐429 score and demonstrated its robustness across technologies and cohorts treated at different institutions.

### 3.2 miR‐200a/b/‐429 is an independent predictive marker of central pelvic recurrence

**Fig. 2:**
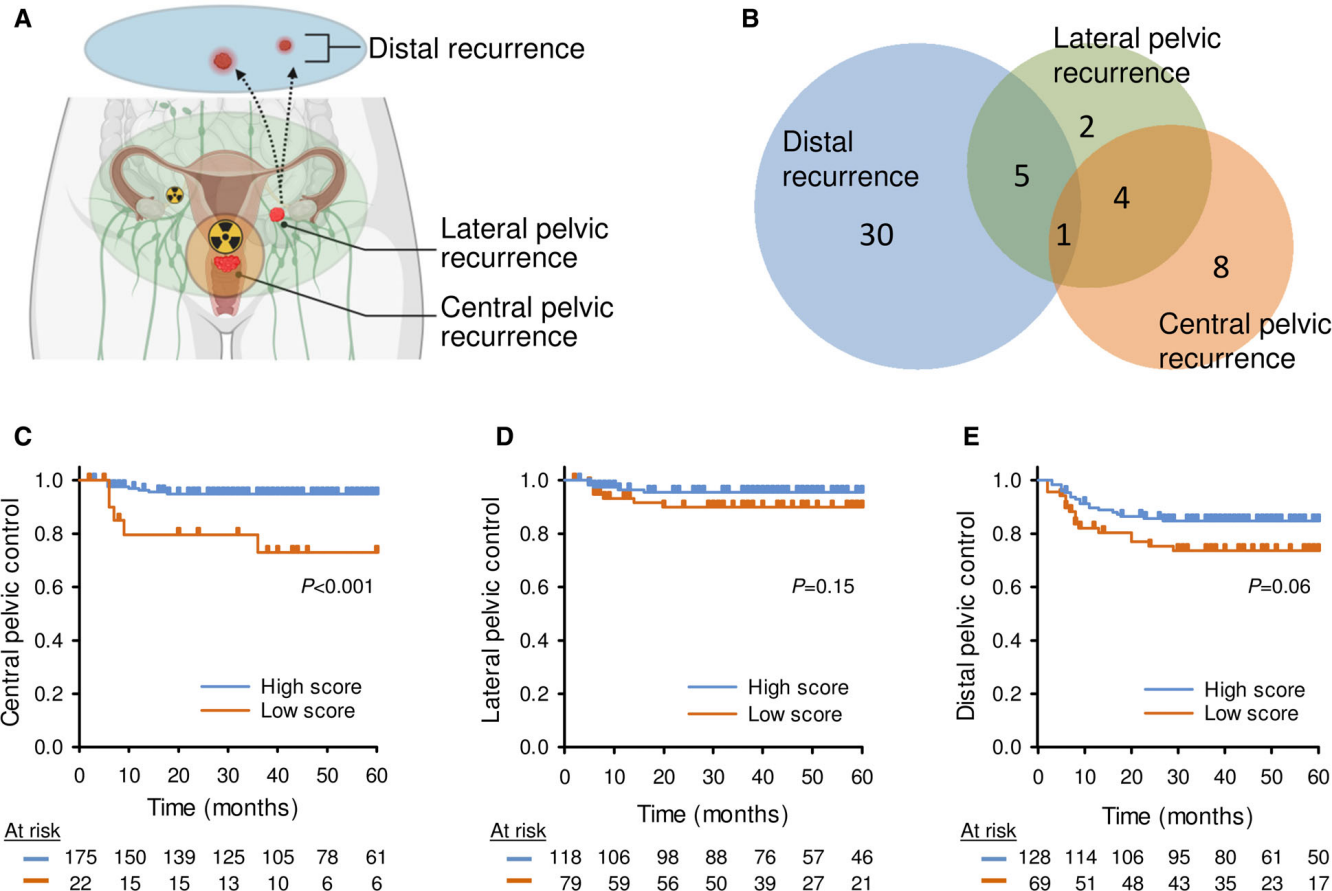
Association between the miR‐200a/b/‐429 score and tumor recurrence at different sites. (A) Schematic illustration of recurrence sites in cervical cancer. Created with BioRender.com. (B) Venn diagram showing site‐specific numbers of recurrences (*n* = 50) in patients of the explorative cohort and validation cohort 1 combined (*n* = 200). Kaplan–Meier curves of patients with high or low miR‐200a/b/‐429 score, using (C) central pelvic control, (D) lateral pelvic control, and (E) distal control as endpoint (*n* = 200). (C–E) 60 months follow up data were used; *P*‐values from log‐rank test and number of patients at risk are indicated; censored data adjusted for recurrence site were used in each analysis; patients were divided into a high and low score group based on the strongest association to the endpoint in each panel.

To identify the site of recurrence that was most strongly associated with the miR‐200a/b/‐429 score, the explorative cohort and validation cohort 1 were merged into a cohort of 200 patients to increase the statistical power. The recurrence sites included central pelvic in 21% of the recurrences, lateral pelvic in 20% and distant metastasis in 59% (Fig. 2A,B). There was a significant correlation between the miR‐200a/b/‐429 score and central recurrence in univariate Cox regression analysis, using continuous score data (*P* = 6.3 × 10^−3^; HR = 0.45; CI 95%, 0.25–0.80; Table 2). Moreover, in log‐rank test, a group of 13% of the patients with the lowest score had a significantly higher risk of central recurrence compared to the others (Fig. 2C). No association with lateral pelvic or distal recurrence was found, neither in univariate Cox analyses on continuous score data (*P* = 3.7 × 10^−1^ and *P* = 3.4 × 10^−1^, respectively; Table 2) or in log‐rank test on patients groups (Fig. 2D,E).

**Table 2:** Cox regression analysis of tumor recurrence at different sites for 200 patients. N.S, nonsignificant.

Factor	Univariate analysis^a^			Multivariate analysis^b^		
	*P*	HR	95% CI	*P*	HR	95% CI
Central pelvic control^c^						
Lymph node status	1.3 × 10^−2^	5.15	1.42–18.73	2.8 × 10^−2^	4.67	1.18–18.54
Tumor volume^d^	(6.3 × 10^−2^)	3.06	0.94–9.97	N.S	–	–
FIGO stage^e^	(1.0 × 10^−1^)	2.56	0.83–7.85	N.S	–	–
Hypoxia status^f^	3.4 × 10^−3^	6.87	1.89–25.00	1.9 × 10^−2^	4.78	1.29–17.74
**miR‐200a/b/‐429 score**	**6.3 × 10^−3^**	**0.45**	**0.25–0.80**	**1.1 × 10^−2^**	**0.43**	**0.23–0.83**
Lateral pelvic control^c^						
Lymph node status	9.9 × 10^−3^	7.37	1.61–33.67	4.8 × 10^−2^	4.81	1.01–22.85
Tumor volume^d^	1.5 × 10^−2^	6.56	1.40–30.00	N.S	–	–
FIGO stage^e^	8.6 × 10^−3^	7.74	2.23–25.84	8.3 × 10^−3^	5.27	1.53–18.15
Hypoxia status^f^	(7.6 × 10^−2^)	2.81	0.89–8.87	N.S	–	–
**miR‐200a/b/‐429 score**	**N.S**	**–**	**–**	**–**	**–**	**–**
Distal control^c^						
Lymph node status	1.1 × 10^−2^	2.39	1.22–4.68	N.S.	–	–
Tumor volume^d^	7.2 × 10^−4^	3.53	1.70–7.32	3.0 × 10^−2^	2.35	1.09–5.08
FIGO stage^e^	2.0 × 10^−6^	5.05	2.61–9.77	1.6 × 10^−4^	3.82	1.90–7.68
Hypoxia status^f^	(6.5 × 10^−2^)	1.85	0.96–3.57	N.S.	–	–
**miR‐200a/b/‐429 score**	**N.S**	**–**	**–**	**–**	**–**	**–**

Results for the miR‐200a/b/‐429 score are highlighted in bold.

^a^ Variables with *P* > 0.05 and ≤ 0.1 in the univariate analysis are shown in parentheses.

^b^ Variables with *P* ≤ 0.1 in the univariate analysis were included in multivariate analysis. The same results were obtained for forward and backward selection.

^c^ Site of recurrence was unknown for three patients.

^d^ Patients were divided into two groups based on the median tumor volume of 36.6 cm^3^.

^e^ Patients were divided into two groups based on FIGO stage IB–IIB or IIIA–IVA.

^f^ Patients were divided into two groups based on a less hypoxic or more hypoxic tumor.

The miR‐200a/b/‐429 score was further related to conventional clinical markers, including FIGO stage, tumor size, and lymph node status, as well as hypoxia status. There was no association between the score and any of these markers (Table S2). In unitivariate Cox regression analysis, lymph node and hypoxia status, in addition to the miR‐200a/b/‐429 score, showed significant association to central pelvic recurrence. Moreover, the miR‐200a/b/‐429 score retained its statistical significance together with lymph node and hypoxia status in multivariate analysis (Table 2). These results suggested a potential of miR‐200a/b/‐429 expression as a biomarker of central pelvic recurrence.

### 3.3 miR‐200a/b/‐429 overexpression increases radiosensitivity in tumor models *in vivo*

To determine whether the miR‐200a/b/‐429 cluster had a direct role in regulation of radiation response, radiation experiments were conducted in cell lines and xenograft tumors with stable overexpression of these candidates. The expression level of each of the five mature miRNA species was successfully increased in the stable mir‐200b‐200a‐429‐GFP transduced SiHa cell line compared to control‐GFP SiHa cell line (Fig. S7A). Protein level of E‐cadherin was higher (*P* = 7.4 × 10^−3^) and cell size smaller (*P* = 1.8 × 10^−3^) in miRNA‐overexpressing cells than in control cells (Fig. S7B–D), in line with the known function of these miRNAs in EMT suppression [37]. miRNA overexpression had no significant effect on cell survival or proliferation rate (Fig. S7E,F), but a minor decrease in fraction of cells in G_1_‐phase of the cell cycle and an increase in S‐phase fraction were seen (Fig. S7G,H). miRNA‐overexpressing tumors (*n* = 23) showed a higher cell density both by histopathology and MRI‐based ADC values compared to control tumors (*n* = 30) (Fig. S8A,B), consistent with the smaller cell size observed *in vitro*. These tumors also had a higher fraction of necrosis, but there was no difference in hypoxic fraction or growth rate (Fig. S8C–F). Our model system therefore seemed to express an EMT‐suppressive phenotype as expected from published work [38], without showing enrichment of radiosensitive features like fraction of cells in G_2_/M phase *in vitro* or oxygenation *in vivo*.

**Fig. 3:**
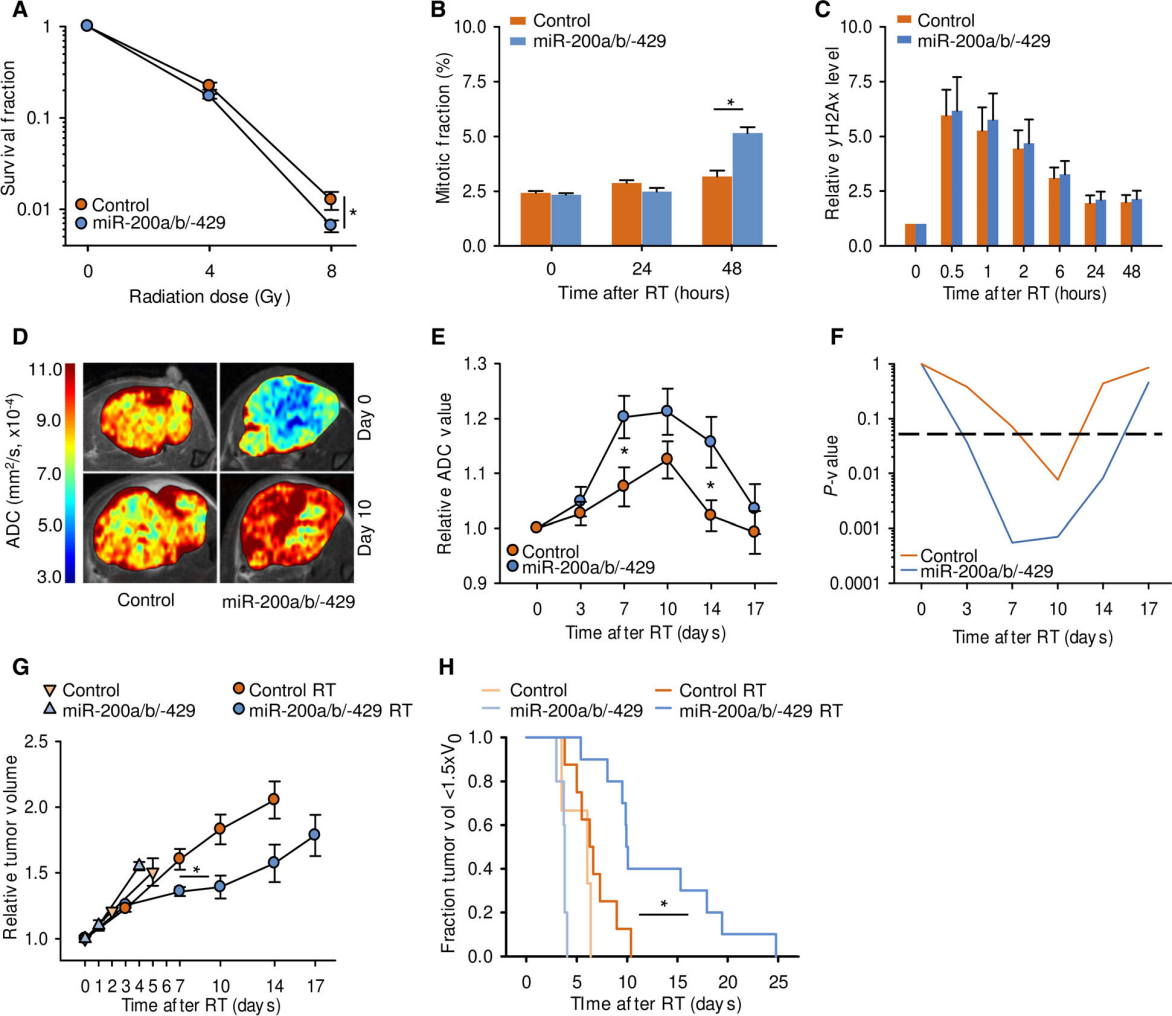
Radiation response of tumor models with stable miR‐200a/b/‐429 overexpression. (A) Clonogenic survival fraction of miRNA‐overexpressing cells and controls after exposure to 4 or 8 Gy, three independent experiments (mean ± SEM; *P < 0.01 from Students *T*‐test). (B) Mitotic fraction in miRNA‐overexpressing cells and controls after exposure to 8 Gy, four independent experiments (mean ± SEM; *P < 0.05 from Student's *T*‐test). (C) Relative γH2Ax level of miRNA‐overexpressing cells and controls after exposure to 8 Gy. Median γH2Ax intensity of each sample are presented relative to nonirradiated cells, four independent experiments (mean ± SEM). No significant difference between groups was observed with Student's *T*‐test. (D) ADC maps of representative tumors before (day 0) and 10 days after exposure to 8 Gy. ADC‐level scale bar is included. (E) ADC in miRNA‐overexpressing tumors (*n* = 10) and controls (*n* = 8) after exposure to 8 Gy. Data relative to ADC at day 0 are shown (mean ± SEM; *P < 0.05 from Students *T*‐test). (F) Significance level (*P*‐values) of the relative change in ADC from day 0 in miRNA‐overexpressing tumors and in controls based on the data in (E) (one sample Student's *T*‐test; stapled line, significance level *P* = 0.05). (G) Tumor volume of miRNA‐overexpressing xenograft tumors (4 nonirradiated, 10 irradiated) and controls (3 nonirradiated, 8 irradiated) after exposure of the irradiated groups to 8 Gy. Data relative to volume at day 0 are shown (mean ± SEM; *P = 0.01 from Student's *T*‐test on *T*_1.5×_, irradiated miRNA‐overexpressing tumors *versus* controls). (H) Kaplan–Meier curves of the tumors shown in (G), using fraction of tumors with a volume below 1.5 times the baseline volume (day 0). The Holm–Sidak method was used for all pairwise multiple comparisons (*P < 0.05).

Radiation treatment (RT) of cell lines with 8 Gy led to a larger decrease in clonogenic survival fraction in miRNA‐overexpressing cells than in control cells (*P* = 1.4 × 10^−2^) (Fig. 3A). The difference was small, but was consistent with a higher fraction of mitotic cells for the miRNA‐overexpressing cells at 48 h after irradiation, and therefore presumably a larger extent of mitotic catastrophe in these cells (Fig. 3B). Both miRNA‐overexpressing cells and controls showed, however, the same elevated G_2_/M‐fraction at 48 h and a considerable arrest in these cell cycle phases (Fig. S9A,B). Moreover, no difference was found in the kinetics of DSB repair between miRNA‐overexpressing cells and controls, as judged from the γH2Ax levels (Fig. 3C). Thus, miR‐200a/b/‐429 overexpression led to only a small increase in cellular radiosensitivity and no detectable effect on DSB repair capacity *in vitro*.

In xenograft tumors, the radiosensitizing effect of miR‐200a/b/‐429 overexpression appeared to be more prominent. The miRNA‐overexpressing tumors showed a significantly higher and more persistent increase in ADC after RT with 8 Gy compared to control tumors (*P* < 5.0 × 10^−2^; Fig. 3D–F), reflecting a greater loss of cellularity and development of necrosis [39, 40]. Moreover, RT led to a significant larger growth delay in these tumors than in irradiated controls (*P* = 1.3 × 10^−2^; Fig. 3G, Table S3). The radiation response enhancement factor (EF) by miR‐200a/b/‐429 overexpression was found to be as high as 6.6 (Table S3). RT caused a marginal, nonsignificant growth delay in control tumors (Fig. 3G), showing that a dose of 8 Gy had minor effect in these tumors with originally low miR‐200a/b/‐429 expression. The increased radiation response in miRNA‐overexpressing tumors was also evident when Kaplan–Meier curves were compared between the experimental groups (*P* = 1.9 × 10^−2^; Fig. 3H). Altogether, although miR‐200a/b/‐429 overexpression had no large effect on radiosensitivity *in vitro*, it seemed to significantly increase radiosensitivity *in vivo*. This supported our hypothesis that low miR‐200a/b/‐429 expression in tumors is associated with increased radioresistance.

### 3.4 miR‐200a/b/‐429 target genes show enrichment of processes involving interactions with ECM

**Fig. 4:**
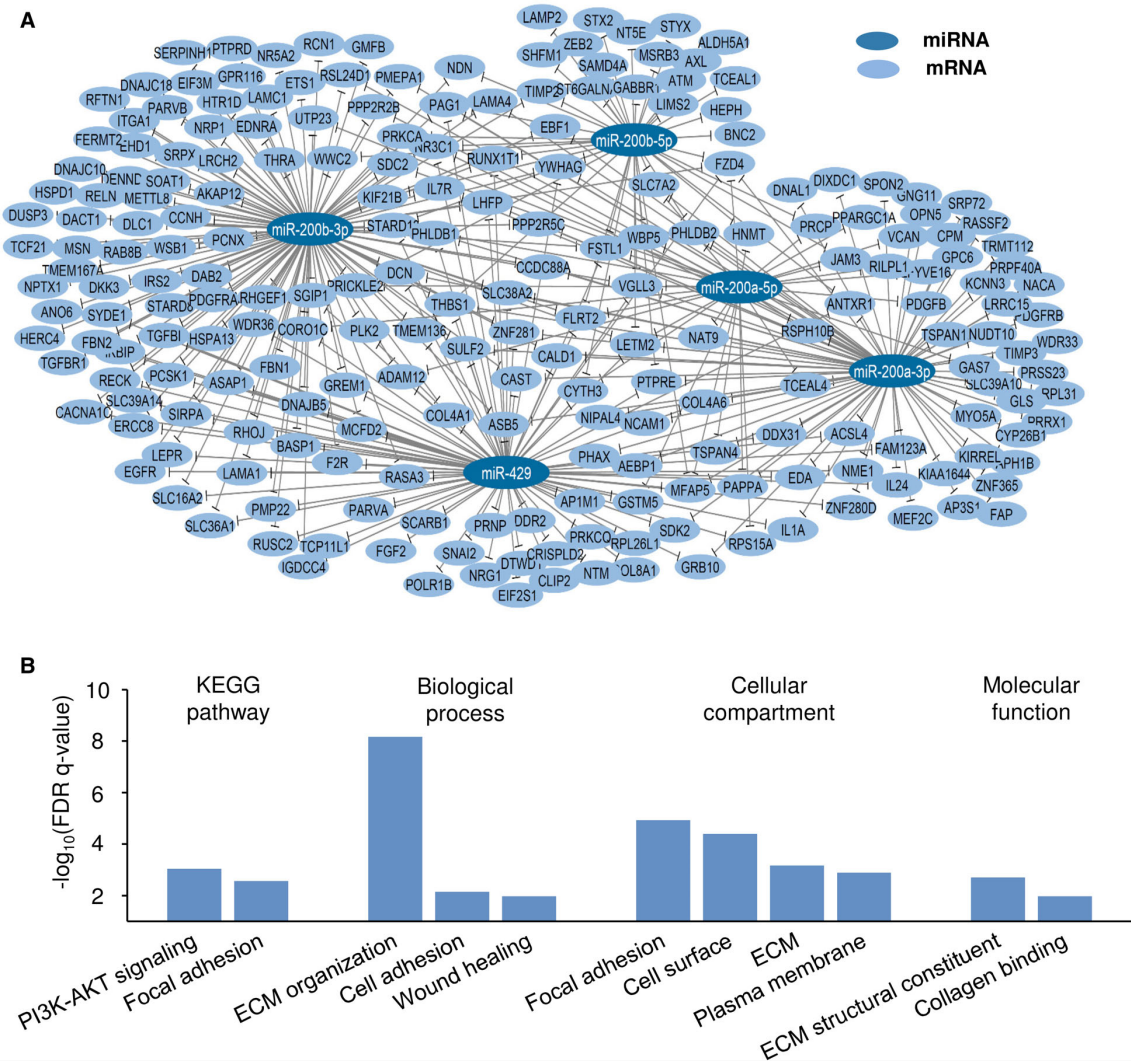
Regulatory network and biological processes associated with miR‐200a/b/‐429. (A) miR‐200a/‐b/‐429 regulatory network. Each miRNA in the miR‐200a/‐b/‐429 cluster is used as node. The interaction partners are their potential target genes, identified from correlation analysis of miRNA and gene expression in the explorative cohort (*n* = 90) and validation cohort 1 (*n* = 110). The network was visualized using cytoscape software v3.6.1. (B) Enriched KEGG pathways and GO terms in the list of 225 potential miR‐200a/b/‐429 target genes. *Y*‐axis denotes significance level from the functional annotation tool david v6.8.

To better understand the role of miR‐200a/b/‐429 as regulators of radiation response in patients, we evaluated potential target genes of the miRNAs. In total 7613 unique, validated or predicted target genes of miR‐200a‐5p/‐3p, 200b‐5p/‐3p, and/or miR‐429 were collected from the miRGate database. Out of these, 225 genes showed significant inverse correlation between gene and miRNA expression in both the explorative cohort and validation cohort 1 (Table S4). They were therefore considered as potential target genes in our patient cohorts. The strong inverse correlation implied that these genes were upregulated in tumors with low miRNA expression. The correlation data in Table S4 were used to construct a regulatory network with the miRNAs as nodes and their target genes as interaction partners. We used a cutoff FDR *q*‐value of 0.1 to identify interactions; that is pairs of miRNA and target gene, for the network. The network visualized pronounced overlap in the target genes of the five miRNAs, where 98 out of 225 (43%) genes had an interaction with more than one miRNA (Fig. 4A). Moreover, each miRNA had more than 30 potential target genes (range 31–118).

Gene ontology and KEGG pathway analysis of the list of all 225 potential target genes was further performed to identify biological processes, cellular compartments, and molecular functions that were enriched, using the global human transcriptome as background. Several interconnected pathways and processes appeared significant, including focal adhesion, ECM organization and the PI3K‐AKT pathway (Fig. 4B; Table S5A). In line with this result, cellular compartments and molecular functions related to ECM were enriched. The list of all 7613 miRGate registered, candidate target genes for the five miRNAs contained genes involved in a large variety of biological processes. A GO and KEGG pathway analysis of the 225 potential target genes was also performed by using these 7613 genes as background. The analysis led to similar results as those presented in Fig. 4B (Table S5B). Downregulation of miR‐200a/b/‐429 therefore seemed to be associated with cell‐ECM interactions and activation of PI3K‐AKT signaling. Moreover, these biological processes represented a specific part of the repertoire of processes controlled by miR‐200a/b/‐429.

## 4 Discussion

Our study identifies a role of the miR‐200a/b/‐429 cluster in tumor radioresistance of cervical cancer. By assessing one of the largest cohorts of patients to date with paired miRNA and site‐specific recurrence data, we showed that miR‐200a/b/‐429 had prognostic impact that was mainly caused by an association with central pelvic recurrence. A unique data set of hypoxia status was also available for all patients and showed that this radioresistance mechanism was not involved. Tumor models with stable miR‐200a/b/‐429 expression were developed for proof‐of‐principle studies, which validated a role of the miRNAs in radiation response. Further, to verify functional activity of miRNAs and understand which pathways they regulate, analysis of their target genes in patient tumors is essential. By assessing global gene expression data of all patients, we addressed this in an explorative manner that helped to propose the resistance mechanisms involved. Our results encourage development of miR‐200a/b/‐429 as a diagnostic biomarker, identifying patients in need for radiosensitizing therapy.

Expression of mature miRNAs derived from both the 5′ and 3′ arm of the miR‐200a and miR‐200b precursor duplex, in addition to miR‐429, was associated with cancer progression in our survival analyses. Moreover, potential target genes were identified for all five members. It is therefore likely that all of them, including the 5p/3p pairs, are functionally active in cervical cancer and regulate different genes, in accordance with reports on 5p/3p pairs of other miRNAs in cancer cells [41, 42]. In addition, all members showed considerable variation in expression level across patients, which is a requirement for further biomarker development [43]. Although highly co‐expressed, all five members were therefore considered in our work. In contrast, expression of miR‐141 or miR‐200c seemed to be less important for chemoradiotherapy resistance in cervical cancer.

Our data suggested that miR‐200a/b/‐429 is associated with treatment resistance locally in the tumor, and not with development of metastases. This hypothesis was further supported by radiation experiments in tumor models. It should be noted, however, that downregulation of miR‐200a/b/‐429 also might increase resistance to the concurrent cisplatin, as indicated for miR‐200b in experimental studies of ovarian cancer [44]. The miR‐200a/b/‐429 target genes were shown to be involved in ECM organization and cell adhesion processes. Such EMT processes are known to be regulated by these miRNAs [11], and are key mechanisms for maintaining a mesenchymal phenotype in cancer cells [45]. Moreover, cancer cell's interaction with ECM has been associated with radioresistance in several cancer types, a mechanism referred to as cell adhesion‐mediated radioresistance [14, 15, 46]. Involvement of this resistance mechanism in our patients is likely. Hence, it was supported by experimental studies, showing a larger radiosensitizing effect of miR‐200a/b/‐429 overexpression in tumors compared to cell lines. Thus, the effect in cell lines was minor and much less than the radiosensitizing effect of for example oxygen in SiHa cells, where the cell death fraction after 8 Gy of irradiation is 10‐fold higher under normoxic than hypoxic conditions [47]. EMT has been implicated in chemotherapy resistance in previous work on cervical cancer, but only few studies have addressed its importance for radioresistance [48]. Our work therefore revealed a novel radioresistance mechanism in this disease that appears to involve interactions between cancer cells and ECM and is mediated by miR‐200a/b/‐429 downregulation.

A limitation of our experimental studies was the use of a single model system. However, the experiments were guided by findings in our patient material, and aimed to provide a proof‐of‐principle regarding radiation response. Moreover, the model was tested both *in vitro* and *in vivo*. Our conclusions are therefore most likely sound and robust with the use of a single model system.

Several downstream pathways mediated by EMT and cell adhesion can be involved in the development of radioresistance. We found enrichment of genes in the PI3K‐AKT pathway among the potential miR‐200a/b/‐429 target genes in patients, indicating that this pathway is important. Downregulation of the miR‐200 family has been associated with activation of this pathway in other cancer types, promoting cell proliferation and survival and regulating intracellular pathways in tumor invasion [49]. Moreover, mutation of pathway member PI3KCA and phosphorylation of AKT have been associated with poor prognosis in cervical cancer [50, 51, 52, 53, 54], showing that PI3K‐AKT is important in progression of this disease. Other pathways controlled by miR‐200a/b/‐429 may, however, also influence radiosensitivity, like focal adhesion‐mediated chromatin modulation via the cytoskeleton [55].

Tumor hypoxia was explored in our work as a potential mechanism underlying downregulation of miR‐200a/b/‐429. This hypothesis was based on studies in cell lines from breast cancer, colorectal cancer and in human endothelial cells [16, 17, 18]. However, our studies on both patient material and model system indicated that this regulation mechanism is not important in cervical cancer. Other mechanisms like epigenetic modulation of the miR‐200a/b/‐429 cluster have been demonstrated in some cancer types [56], and should be investigated. Analysis of tumor hypoxia against our site‐specific recurrence data further showed that its correlation with locoregional control, as reported in previous work [19] mainly reflected an effect on central pelvic recurrence. Hence, we here propose that at least two independent tumor radioresistence mechanisms are important in cervical cancer, one involving downregulation of miR‐200a/b/‐429 and another hypoxia.

The association between the miR‐200a/b/‐429 score and central pelvic recurrence was independent of conventional clinical markers. The expression level of this miRNA cluster may therefore contain information about tumor radioresistance that is not covered by current diagnostics. This encourages development of a diagnostic test for patients referred to chemoradiotherapy, measuring miR‐200a/b/‐429 expression. Decision about the optimal test assay, definition of a cutoff score for classifying patients to the low expression group, and establishment of an internal control would be important aspects of such development. An RT‐qPCR kit, which includes reference miRNAs with stable expression in tumor biopsies [24], could be an appropriate low‐cost approach. It should also be considered whether a refined set of miRNAs would improve the specificity and sensitivity of the test. Moreover, investigations of whether the miRNAs are detectable in blood or plasma and their potential as a blood based test would be of considerable interest. Successful establishment of a test would help to select patients for clinical trials on strategies to overcome tumor resistance, and to exclude patients with no expected benefit. Several exciting strategies are upcoming, like combination therapy with radiation and targeted radiosensitizers, including inhibitors of the PI3K‐AKT pathway and of cell–matrix interactions [57], or escalation of the radiation dose within normal‐tissue tolerance by proton therapy [58].

## 5 Conclusion

We have identified miR‐200a/b/‐429 as an independent predictor of central pelvic recurrence in cervical cancer and a promising candidate biomarker for tumor radioresistance. Downregulation of miR‐200a/b/‐429 represents a new radioresistance mechanism that operates independently of hypoxia and seems to involve interaction between cancer cells and ECM.

## Conflict of interest

The authors declare no conflict of interest.

## Author contributions

AN and HL conceived and designed the project. AN, THi, VES, E‐KA, TSE, and BG acquired the data and performed the experiments. AN, THi, THo, VES, and TSE analyzed the data and interpreted the results. CSF, THo, GBK, and TS provided resources and data curation. AN and HL wrote and edited the paper with input from all other authors.

## Supporting information


**Fig. S1.** Representative example showing gating of cell subpopulations.
**Fig. S2.** IHC‐staining of xenograft tumor sections.
**Fig. S3.** Definition of necrotic and hypoxic areas in xenograft tumor sections.
**Fig. S4.** Cell segmentation.
**Fig. S5.** Expression of the miR‐200 family in cervical cancer.
**Fig. S6.** Heteroscedasticity analysis.
**Fig. S7.** *In vitro* characterization of miR‐200a/b/‐429‐overexpressing SiHa cells.
**Fig. S8.** *In vivo* characterization of miR‐200a/b/‐429‐overexpressing SiHa tumors.
**Fig. S9.** Radiation effect on cell cycle distribution in miR‐200a/b/‐429‐overexpressing SiHa cells.

**Table S1.** Patient characteristics.
**Table S2.** miR‐200a/b/‐429 expression score *versus* clinical markers and hypoxia status for 200 patients.
**Table S3.** Tumor growth delay (TDG) in miR‐200a/b/‐429‐overexpressing xenografts after radiation treatment (RT).

**Table S4.** Spearman correlation between miRNA and gene expression for the potential target genes of miR‐200a/b/‐429.

**Table S5.** GO and KEGG enrichment analyses.
