# Supplementary material for: miR‐200a/b/‐429 downregulation is a candidate biomarker of tumor radioresistance and independent of hypoxia in locally advanced cervical cancer
Source: Mol Oncol. 2022 Feb 15;16(6):1402–19. doi: 10.1002/1878-0261.13184 (PMC8936520; doi:10.1002/1878-0261.13184)
Supplement: Supplementary file 1 — Fig. S1. Representative example showing gating of cell subpopulations. Fig. S2. IHC‐staining of xenograft tumor sections. Fig. S3. Definition of necrotic and hypoxic areas in xenograft tumor sections. Fig. S4. Cell segmentation. Fig. S5. Expression of the miR‐200 family in cervical cancer. Fig. S6. Heteroscedasticity analysis. Fig. S7. In vitro characterization of miR‐200a/b/‐429‐overexpressing SiHa cells. Fig. S8. In vivo characterization of miR‐200a/b/‐429‐overexpressing SiHa tumors. Fig. S9. Radiation effect on cell cycle distribution in miR‐200a/b/‐429‐overexpressing SiHa cells. [file MOL2-16-1402-s001.pdf]

Supplementary Figures S1-S9 for

miR-200a/b-429 downregulation is a candidate biomarker of tumor radioresistance and independent of hypoxia in locally advanced cervical cancer

Anja Nilsen[1*], Tiril Hillestad[2],Vilde E. Skingen[1], Eva-Katrine Aarnes[1], Christina S. Fjeldbo[1], Tord Hompland[1,2], Tina Sandø Evensen[2], Trond Stokke[2], Gunnar B. Kristensen[3,4], Beata Grallert[1] and Heidi Lyng [1,5*]

[1]Department of Radiation Biology, Norwegian Radium Hospital, Oslo University Hospital, Oslo, Norway. [2]Department of Core Facilities, Norwegian Radium Hospital, Oslo University Hospital, Oslo, Norway. [3]Department of Gynecological Oncology, Norwegian Radium Hospital, Oslo University Hospital, Oslo, Norway. [4]Institute of Cancer Genetics and Informatics, Oslo University Hospital, Oslo, Norway, [5]Department of Physics, University of Oslo, Oslo, Norway.

*Corresponding Authors:

Heidi Lyng, Department of Radiation Biology, Norwegian Radium Hospital, Oslo University Hospital, Pb 4950 Nydalen, Oslo 0424, Norway. Phone: 472-293-1478; E-mail: heidi.lyng@rr-research.no

Anja Nilsen, Department of Radiation Biology, Norwegian Radium Hospital, Oslo University Hospital, Pb 4950 Nydalen, Oslo 0424, Norway. Phone: 472-278-1463; E-mail: anja.nilsen@rr-research.no

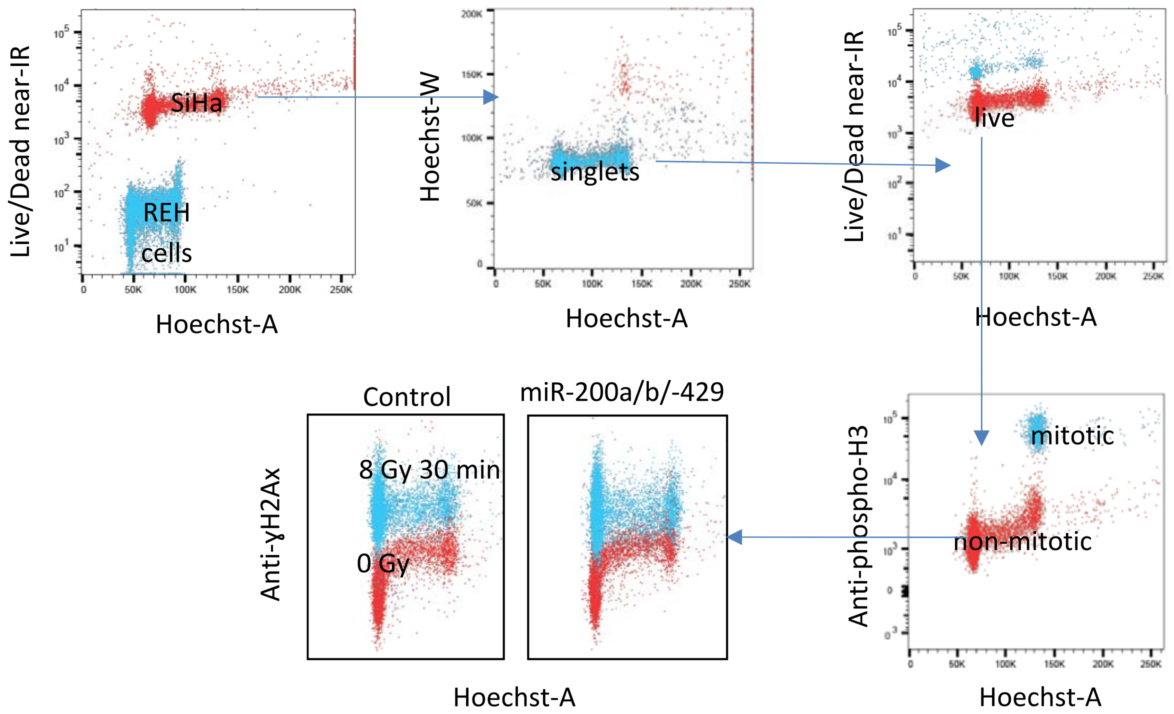

Supplementary Figure S1. **Representative example showing gating of cell subpopulations**. **Left upper panel**: SiHa cells (miR-200a/b/-429 transduced or control) and REH cells were gated on positive/negative Live/Dead near-IR staining (y-axis) and DNA content (Hoechst A, x-axis**). Middle upper panel**: Cell singlets were gated on DNA content (Hoechst-W and Hoechst-A). **Right upper panel**: Live cells were gated on Live/Dead near-IR intensity (y-axis). **Right lower panel**: Mitotic fraction was gated on anti-phospho-histone H3 intensity (y-axis) and DNA content (x-axis) and excluded from analysis of γH2Ax intensities due to high background of γH2Ax levels in mitotic cells. **Left lower panel**: The non-mitotic cell population was used in γH2Ax intensity calculations (median values).

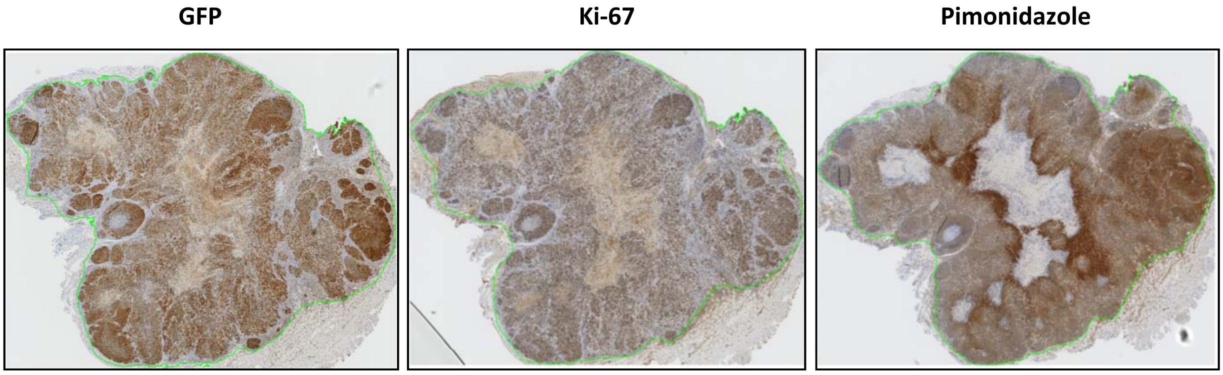

Supplementary Figure S2. IHC-staining of xenograft tumor sections. Example of IHC-stained tumor sections with anti-GFP, anti-Ki-67 and anti-pimonidazole (brown). Hematoxylin (blue) used as counterstain. ROI is marked by the green line.

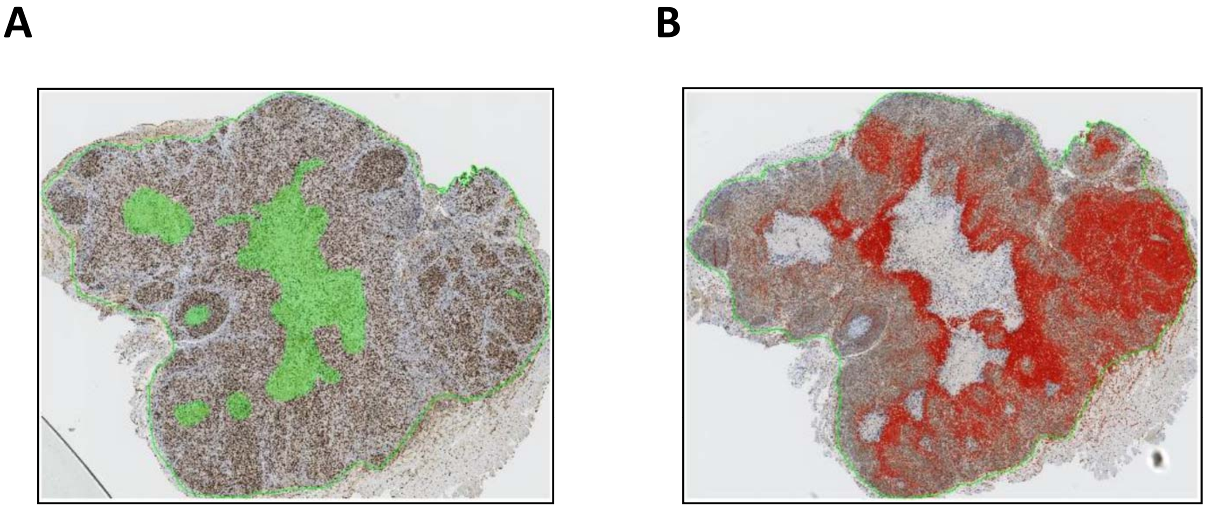

Supplementary Figure S3. **Definition of necrotic and hypoxic areas in xenograft tumor sections**. Representative RGB images of areas defined as **A**) necrotic (green) in Ki-67-stained tumor section and **B**) hypoxic (red) in pimonidazole-stained tumor section.

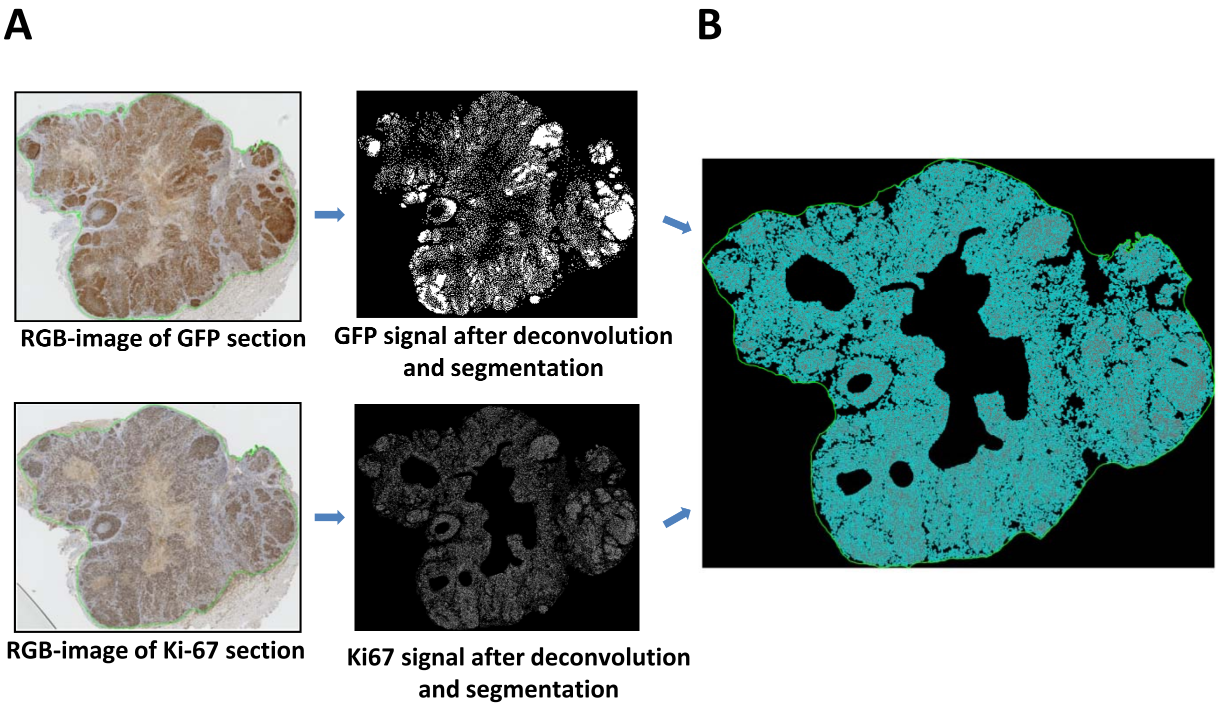

Supplementary Figure S4. **Cell segmentation**. **A**) Representative DAB and binary images of GFP-stained (upper panel) and Ki-67-stained (lower panel) tumor sections. **B**) Defined areas included after merged image processing (red: Ki-67; green: GFP).

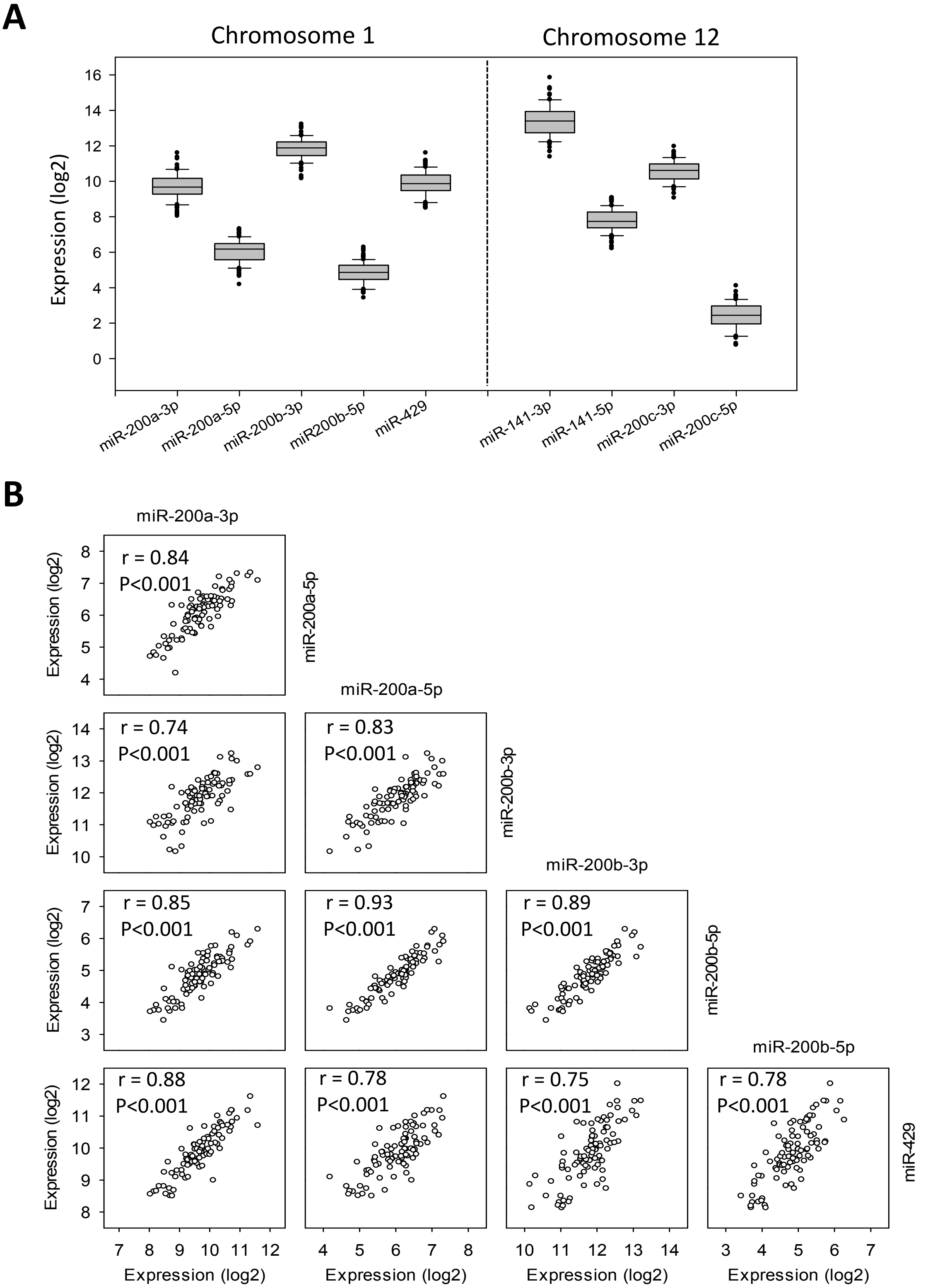

Supplementary Figure S5. **Expression of the miR-200 family in cervical cancer. A**) Expression level (RPM, log_2) from small RNA sequencing data for each of the nine mature miRNAs located in miR-200 clusters on chromosome 1 (left) and chromosome 12 (right) in the explorative cohort (n=90). Box plots represent median, quartiles, range and outliers (circles). **B**)Correlations of miR-200a-5p/-3p, miR-200b-5p/-3p and miR-429 expression (RPM, log2) from small RNA sequencing data of the explorative cohort (n=90). Pearson correlation coefficients (r) and *P*-values indicated.

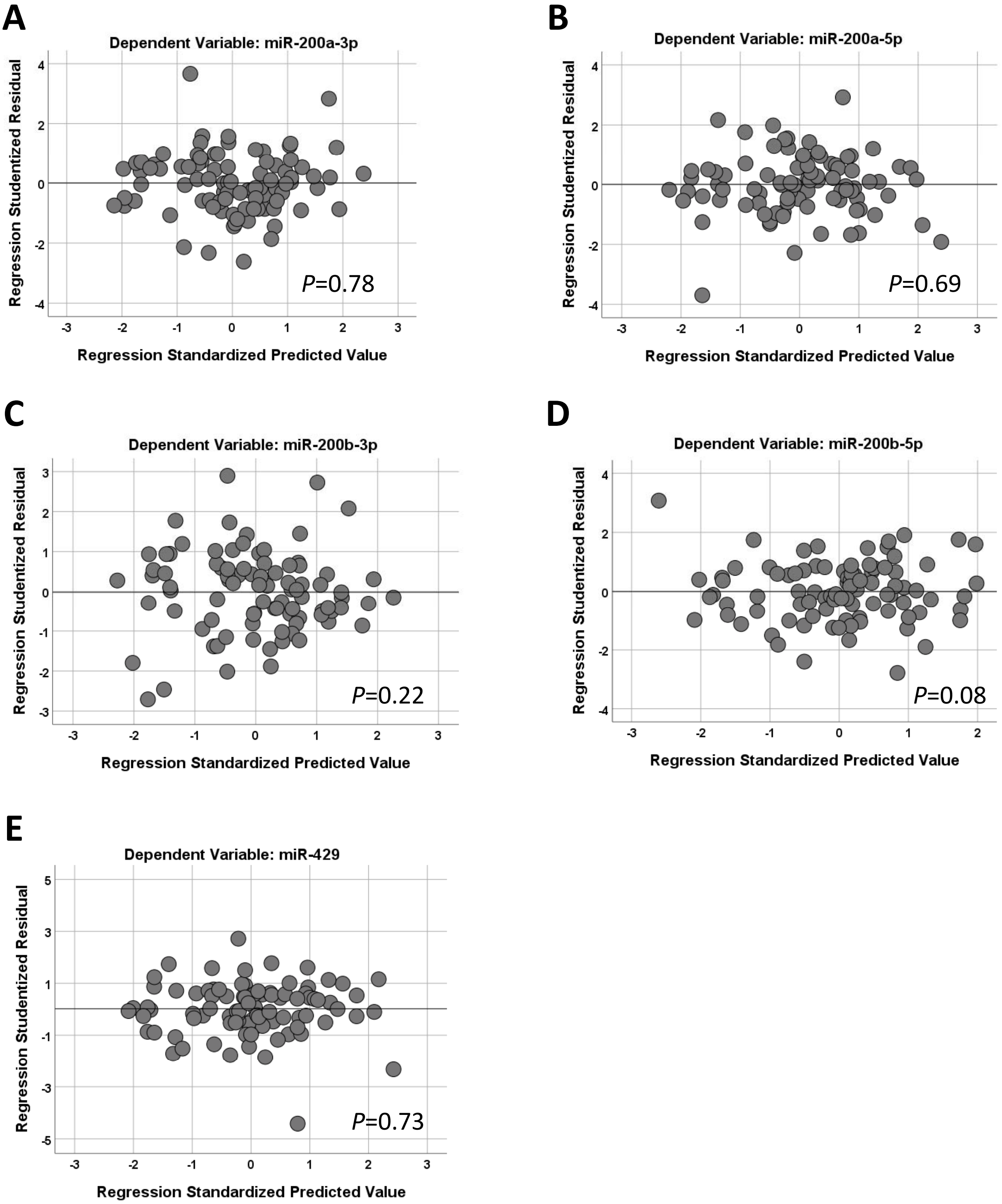

Supplementary Figure S6: Heteroscedasticity analysis. Heteroscedasticity plots of the standardized residuals of (**A**) miR-200a-3p, (**B**) miR-200a-5p, (**C**) miR-200b-3p, (**D**) miR-200b-5p and (**E**) miR-429 *versus* the predictive values of the standardized independent variables (the remaining miRNAs in the linear regression model). *P*-values from White test are indicated.

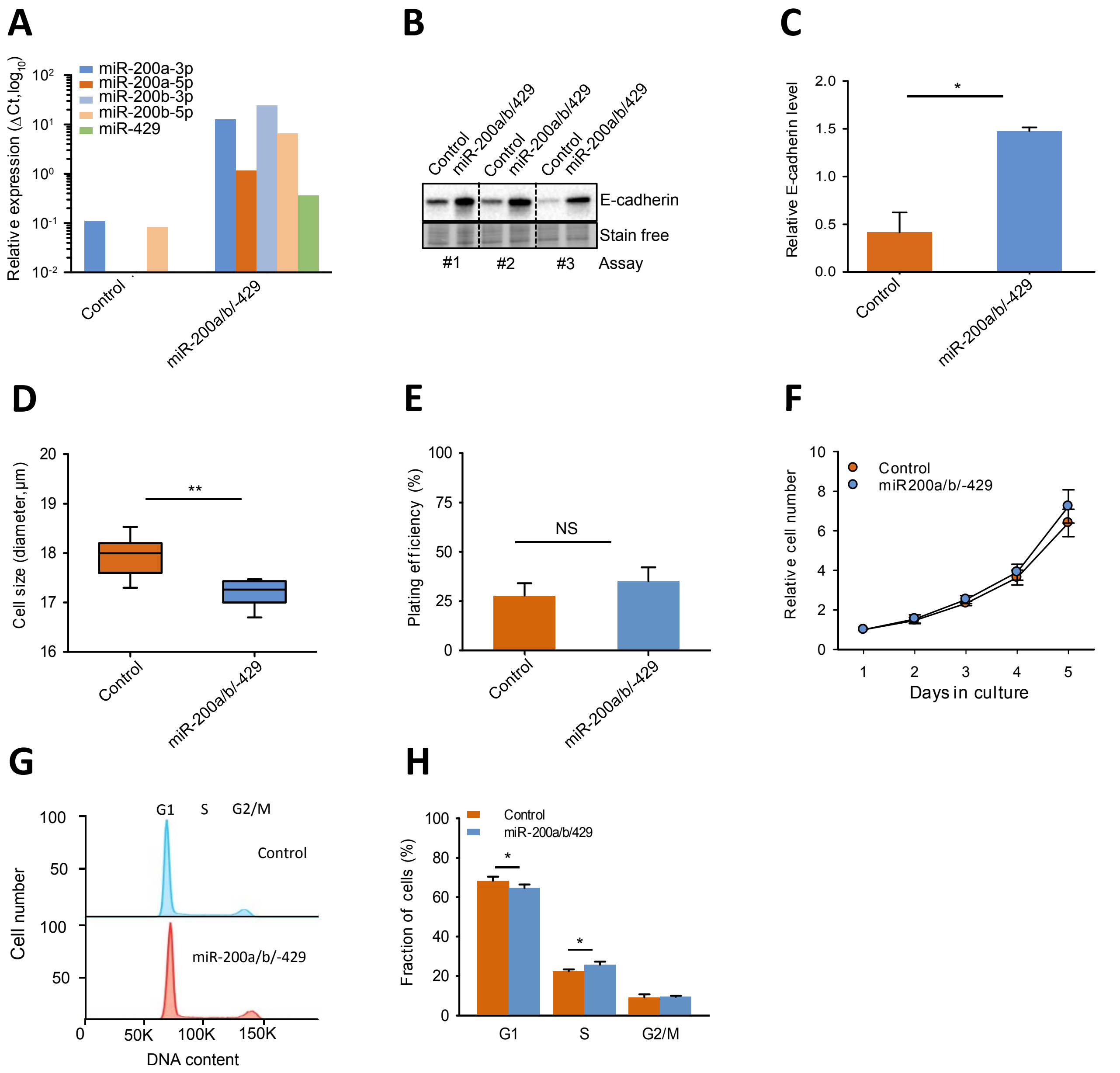

Supplementary Figure S7. *In vitro* characterization of miR-200a/b/-429-overexpressing SiHa cells. **A)** Relative expression (ΔCt) by RT-qPCR of the five mature miRNAs in the miR-200a/b-429 cluster in control cells and miR-200a/b/-429-overexpressing SiHa cells. **B)** Western blot of E-cadherin protein level of control cells and miR-200a/b/-429-overexpressing cells in total protein extracts from three independent assays. Total protein was used as loading control and detected with UV-activation of stain free blots. **C)** Quantification of E-cadherin protein level relative to total protein for the three samples shown in B (mean±SD, right). **D**) Cell diameter of control cells and miR-200a/b/-429-overexpressing cells in suspension. Box plots represent median cell diameter (µm) in samples from seven independent experiments. **E)** Quantification of plating efficiency in clonogenic assays of control cells and miR-200a/b/-429-overexpressing cells from three independent experiments (mean±SD). **F)** Relative cell number *versus* time of cultured control cells and miR-200a/b/-429-overexpressing cells. Cell number relative to number of cells at day 1 after plating in three independent assays are shown (mean±SEM). **G**) Representative histograms of DNA content and cell cycle distribution from flow cytometry analysis of control cells and miR-200a/b/-429-overexpressing cells. **H**) Fraction of control cells and miR-200a/b/-429 overexpressing cells in G_1, S and G_2/M cell cycle phase. Data from four independent experiments (mean±SEM). Students T-test or Mann-Whitney U-test; *, P<0.05; **, P<0.005.

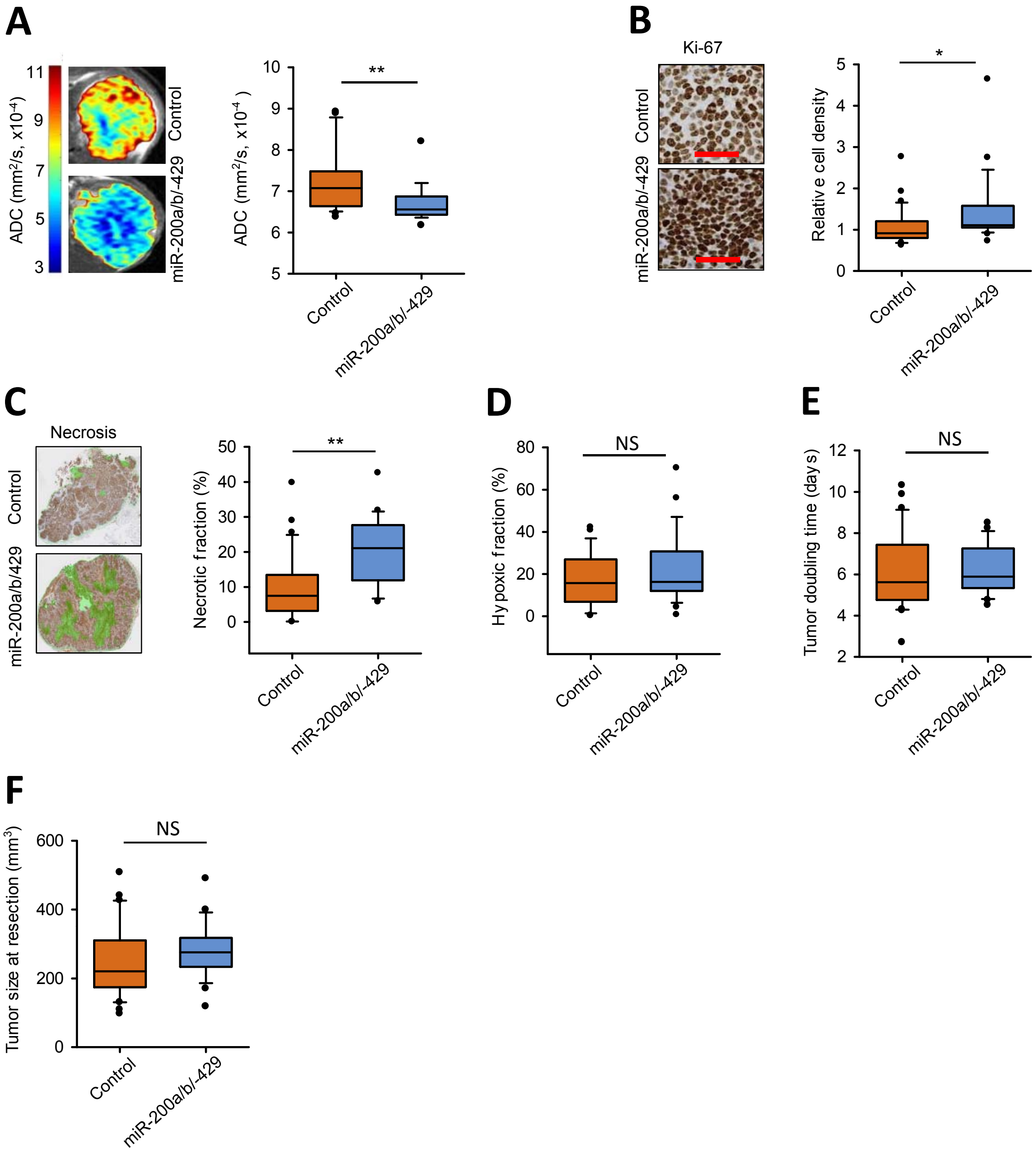

Supplementary Figure S8. *In vivo* characterization of miR-200a/b/-429-overexpressing SiHa tumors. **A**) DW-MRI assessment of cell density in control tumors and miR-200a/b/-429-overexpressing tumors. ADC maps of a representative tumor of each group with color coded scale bar of ADC-values included (left) and box plot of median ADC-values in each group (right). Non-physiological ADC-values were not included in the analysis. **B**) Cell density in control tumors and miR-200a/b/-429-overexpressing tumors. Ki-67 positive cells (brown) in representative tumor sections from each group (scale bar=60 um, left), and box plot showing number of Ki-67 positive cells relative to GFP-positive parenchyma region in co-registered tumor sections (right). **C)** Necrosis in control tumors and miR-200a/b/-429-overexpressing tumors. Necrotic regions (green) in representative tumor sections from each group (left), and box plot of necrotic fraction relative to the entire tumor region (right). **D**) Hypoxic fraction in control tumors and miR-200a/b/-429 overexpressing tumors. Box plot of pimonidazole-positive fraction relative to the entire tumor region. Necrotic regions defined in co-registered tumor sections were excluded from analysis. **E**) Tumor doubling time (days) and **F**) tumor volume at resection in control and miR-200a/b/-429-overexpressing tumors assessed by T_2W-MRI. A-F), Box plots represent median, quartiles, range and outliers (circles); Students T-test or Mann-Whitney U-test statistics, $**P < 0.005$ NS, not significant; n=30 control tumors; n=23 miR-200a/b/-429-overexpressing tumors.

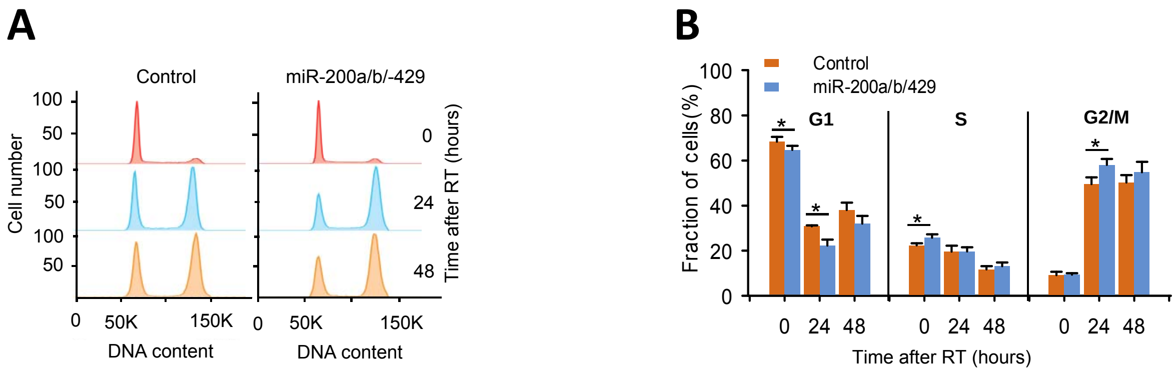

Supplementary Figure S9. Radiation effect on cell cycle distribution in miR-200a/b/-429-overexpressing SiHa cells. **A**) Representative histograms of DNA content and cell cycle distribution from flow cytometry assays of control cells and miR-200a/b/-429-overexpressing cells analyzed before (0 hours) and at indicated timepoints after exposure to 8 Gy. **B)** Fraction of control cells and miR-200a/b/-429 overexpressing cells in G_1, S and G_2/M cell cycle phase analyzed before (0 hours) and at indicated timepoints after exposure to 8 Gy. Data from four independent experiments (mean±SEM). Students T-test; *, P<0.05.
