# Supplementary material for: miR‐200a/b/‐429 downregulation is a candidate biomarker of tumor radioresistance and independent of hypoxia in locally advanced cervical cancer
Source: Mol Oncol. 2022 Feb 15;16(6):1402–19. doi: 10.1002/1878-0261.13184 (PMC8936520; doi:10.1002/1878-0261.13184)
Supplement: Supplementary file 2 — Table S1. Patient characteristics. Table S2. miR‐200a/b/‐429 expression score versus clinical markers and hypoxia status for 200 patients. Table S3. Tumor growth delay (TDG) in miR‐200a/b/‐429‐overexpressing xenografts after radiation treatment (RT). [file MOL2-16-1402-s003.pdf]

Supplementary Tables S1-S3 for

miR-200a/b-429 downregulation is a candidate biomarker of tumor radioresistance and independent of hypoxia in locally advanced cervical cancer

Anja Nilsen[1*], Tiril Hillestad[2],Vilde E. Skingen[1], Eva-Katrine Aarnes[1], Christina S. Fjeldbo[1], Tord Hompland[1,2], Tina Sandø Evensen[2], Trond Stokke[2], Gunnar B. Kristensen[3,4], Beata Grallert[1] and Heidi Lyng [1,5*]

[1]Department of Radiation Biology, Norwegian Radium Hospital, Oslo University Hospital, Oslo, Norway. [2]Department of Core Facilities, Norwegian Radium Hospital, Oslo University Hospital, Oslo, Norway. [3]Department of Gynecological Oncology, Norwegian Radium Hospital, Oslo University Hospital, Oslo, Norway. [4]Institute of Cancer Genetics and Informatics, Oslo University Hospital, Oslo, Norway, [5]Department of Physics, University of Oslo, Oslo, Norway.

*Corresponding Authors:

Heidi Lyng, Department of Radiation Biology, Norwegian Radium Hospital, Oslo University Hospital, Pb 4950 Nydalen, Oslo 0424, Norway. Phone: 472-293-1478; E-mail: heidi.lyng@rr-research.no

Anja Nilsen, Department of Radiation Biology, Norwegian Radium Hospital, Oslo University Hospital, Pb 4950 Nydalen, Oslo 0424, Norway. Phone: 472-278-1463; E-mail: anja.nilsen@rr-research.no

Supplementary Table S1. Patient characteristics

Characteristics	miRNA explorative cohort (n=90) No	%	miRNA validation cohort 1 (n=110) No	%	miRNA validation cohort 2 (n=79)* No	%	p-value [g]
Age, years							$6.7x10^{-2}$
Median	52.9		54.9		48		
Range	21.8-83.9		23.8-84.2		26-84		
FIGO stage							$2.9x10^{-1}$
1b -2b	70	78	76	69	61	77	
3b-4a	20	22	34	31	18	23	
Tumor size (median)							
Volume (range) [a]	26.8 cm^3 (2.8-266.8)		43.8 cm^3 (1.9-302.4)				$3.1x10^{-3}$
Diameter (range)					5cm (2.3-8)		
NA	6	7	5	5			
Lymph node status [b]							$4.5x10^{-1}$
Positive	44	49	45	41	39	49	
Negative	46	51	65	59	40	51	
Concurrent cisplatin							$5.5x10^{-1}$
Yes (median no. of courses)	74 (5)	82	90 (5)	82	NA		
No	16	18	20	18	NA		
Hypoxia status [c]							$7.5x10^{-2}$
Less hypoxic	63	71	64	58	NA		
More hypoxic	26	29	46	42	NA		
Observation time (months) [d]							$1.08x10^{-11}$
Median	43		60		60		
Range	22-60		28-60		9-60		
Status							
PFS [e]	19	21	34 [f]	31	26	32	$1.8x10^{-1}$
Central pelvic recurrence	5	6	8	7	NA		$7.8x10^{-1}$
Lateral pelvic recurrence	4	4	8	7	NA		$5.6x10^{-1}$
Distal recurrence (metastasis)	11	12	25	23	NA		$9.4x10^{-2}$

FIGO, Federation International de Gynecologie et d'Obstetrique

[a] Calculated based on 3 orthogonal diameters (a,b,c) as (π/6)abc

[b] Detected by MRI or CT at diagnosis, according to the response evaluation criteria in solid tumors (RECIST) v 1.1

[c] The tumors were classified as more or less hypoxic according to the 6-gene hypoxia classifier (Fjeldbo et al, 2016)

[d] Follow up time for patients with no events (recurrences) up to 60 months

[e] Follow up data up to 60 months

[f] Location of recurrence was unknown for three patients

[g] Kruskall-Wallis-, Chi-square-, Fisher exact- or Mann-Whitney U-test were used where appropriate

* How et al, 2014. Training cohort

Supplementary Table S2. miR-200a/b/-429 expression score *versus* clinical markers and hypoxia status for 200 patients.

**Marker**	**Statistical test**	***P*-value**
FIGO stage (I,II,III,IV)	Kruskal-Wallis test	4.2×10^{-1}
Lymph node status (pos. vs neg.)	Mann Whitney U-test	5.5×10^{-1}
Tumor volume (mm^3)	Spearman rho correlation	3.7×10^{-1}
Hypoxia status (more vs less hypoxic)	Mann Whitney U-test	6.4×10^{-1}

Supplementary Table S3. Tumor growth delay (TDG) in miR-200a/b/-429-overexpressing xenografts after radiation treatment (RT)

**Group**	**Tumors (n)**	**V_0 (mm^3) [a] (mean±SEM)**	**$T_{1.5x}$ (mean±SEM)**	**TGD (days) [b] (mean±SEM)**	***P*-value TGD 1.5x [c]**	**Normalized TGD (days) [d] (mean±SEM)**	**Enhancement factor [e]**
Control [f]	3	200±19.2	5.3±0.9	-			
miR-200a/b/-429 [f]	4	233±19.1	3.7±0.2	- 1.6±0.2			
Control + RT	8	210±11.3	6.7±0.8	1.4±0.8			
miR-200a/b/-429 + RT	10	230±11.7	13.0±1.9	7.7±1.9	$1.3x10^{-2}$	9.4±1.9	6.6

[a] V_0, tumor volume at time of radiation (day 0)

[b] TGD = difference in $T_{1.5x}$ when compared to Control

[c] Students T-test, compared to Control + RT

[d] Normalized TGD = tumor growth delay in miR-200 + IR group minus TGD in miR-200 group

[e] Normalized TGD of miR-200a/b/-429 + RT group divided by TGD of Control + RT group

[f] Tumor growth data obtained from non-irradiated tumors in the xenograft characterization study. Tumors which matched the volume V_0 and following 1.5 x V_0 in the corresponding groups of RT-tumors (Control + RT and miR-200a/b/-429 + RT, respectively) were used to enable comparable growth data.
